# Thoracic and cardiovascular surgeries in Japan during 2024: Annual report by the Japanese Association for Thoracic Surgery

**DOI:** 10.1007/s11748-025-02242-y

**Published:** 2026-02-27

**Authors:** Committee for Scientific Affairs, The Japanese Association for Thoracic Surgery, Naoki Yoshimura, Yukio Sato, Hiroya Takeuchi, Tomonobu Abe, Toshio Doi, Toyofumi Fengshi Yoshikawa, Yasutaka Hirata, Michiko Ishida, Hisashi Iwata, Takashi Kamei, Nobuyoshi Kawaharada, Shunsuke Kawamoto, Kohji Kohno, Kazuo Koyanagi, Hiraku Kumamaru, Goro Matsumiya, Kenji Minatoya, Noboru Motomura, Rie Nakahara, Morihito Okada, Hisashi Saji, Aya Saito, Kenji Suzuki, Hirofumi Takemura, Yasue Kimura, Wataru Tatsuishi, Hiroyuki Yamamoto, Akira Shiose, Takushi Yasuda, Masayuki Chida, Hideyuki Shimizu

**Affiliations:** 1 Committee for Scientific Affairs, The Japanese Association for Thoracic Surgery, Tokyo, Japan; 2 Department of Thoracic and Cardiovascular Surgery, Graduate School of Medicine, University of Toyama, Toyama, Japan; 3 Department of Thoracic Surgery, University of Tsukuba, Tsukuba, Japan; 4 Department of Surgery, Hamamatsu University School of Medicine, Shizuoka, Japan; 5 Department of Cardiac Surgery, Fujita Health University Hospital, Aichi, Japan; 6 Department of Thoracic Surgery, Nagoya University Graduate School of Medicine, Nagoya, Japan; 7 Department of Cardiovascular Surgery, National Center for Child Health and Development, Tokyo, Japan; 8 Cardiac Surgery, Handa City Hospita, Aichi, Japan; 9 Department of General Thoracic Surgery, Gifu University Hospital, Gifu, Japan; 10 Department of Surgery, Graduate School of Medicine, Tohoku University, Sendai, Japan; 11 Department of Cardiovascular Surgery, Sapporo Medical University School of Medicine, Sapporo, Japan; 12 Department of Cardiovascular Surgery, Tohoku Medical and Pharmaceutical University Hospital, Sendai, Japan; 13 Department of Gastrointestinal Tract Surgery, Fukushima Medical University, Fukushima, Japan; 14 Department of Gastroenterological Surgery, Tokai University School of Medicine, Isehara, Japan; 15 Graduate School of Data Science, Yokohama City University, Yokohama, Japan; 16 Department of Cardiovascular Surgery, Chiba University Graduate School of Medicine, Chiba, Japan; 17 Department of Cardiovascular Surgery, Graduate School of Medicine, Kyoto University, Kyoto, Japan; 18 Department of Cardiovascular Surgery, Toho University Sakura Medical Center, Chiba, Japan; 19 Division of Thoracic Surgery, Tochigi Cancer Center, Tochigi, Japan; 20 Surgical Oncology, Hiroshima University, Hiroshima, Japan; 21 Department of Chest Surgery, St. Marianna University School of Medicine, Kawasaki, Japan; 22 Department of Surgery, Graduate School of Medicine, Yokohama City University, Yokohama, Japan; 23 Department of General Thoracic Surgery, Juntendo University School of Medicine, Tokyo, Japan; 24 Department of Cardiovascular Surgery, Toyama Red Cross Hospital, Toyama, Japan; 25 Department of Gastroenterological Surgery, National Hospital Organization Kyushu Cancer Center, Fukuoka, Japan; 26 Division of Cardiovascular Surgery, Department of General Surgical Science, Gunma University, Maebashi, Japan; 27 Department of Healthcare Quality Assessment, Graduate School of Medicine, The University of Tokyo, Tokyo, Japan; 28 Department of Cardiovascular Surgery, Kyusyu University, Graduate School of Medicine, Fukuoka, Japan; 29 Department of Surgery, Kindai University, Osaka, Japan; 30 Department of General Thoracic Surgery, Dokkyo Medical University, Tochigi, Japan; 31 Department of Cardiovascular Surgery, Keio University, Tokyo, Japan

Since 1986, the Japanese Association for Thoracic Surgery (JATS) has conducted annual thoracic surgery surveys throughout Japan to determine statistics on the number of procedures performed by surgical categories. Herein, we summarize the results of the association’s annual thoracic surgery surveys in 2024.

Adhering to the norm thus far, thoracic surgery had been classified into three categories, including cardiovascular, general thoracic, and esophageal surgeries, with patient data for each group being examined and analyzed. We honor and value all members’ continued professional support and contributions.

Incidence of hospital mortality was included in the survey to determine nationwide status, which has contributed to Japanese surgeons’ understanding of the present status of thoracic surgery in Japan while helping in surgical outcome improvements by enabling comparisons between their work and that of others. This approach has enabled the association to gain a better understanding of present problems and prospects, which is reflected in its activities and member education.

The 30-day mortality (also known as *operative mortality*) is defined as death within 30 days of surgery, regardless of the patient’s geographic location, including post-discharge from the hospital. *Hospital mortality* is defined as death within any time interval following surgery among patients yet to be discharged from the hospital.

Transfer to a nursing home or a rehabilitation unit is considered hospital discharge unless the patient subsequently dies of complications from surgery, while hospital-to-hospital transfer during esophageal surgery is not considered a form of discharge. In contrast, hospital-to-hospital transfer 30 days following cardiovascular and general thoracic surgeries are considered discharge given that National Clinical Database (NCD)-related data were used in these categories.

The pandemic of the coronavirus disease 2019 (COVID-19) resulted in a global healthcare and financial crisis in December 2019 and by March 2020 [1]. There was a significant estimated reduction in national case volume of cardiovascular, general thoracic, and esophageal surgeries in Japan during 2020 to 2022 [2–6]. The number of surgeries began to increase in 2023 [7], but the impact of population decline has also become a concern. We have to continue the estimation of the nationwide effect of SARS-CoV-2 pandemic and population decline on thoracic surgery in Japan, with surgical volume, outcomes and patient data for each group.

## Survey abstract

All data on cardiovascular, general thoracic, and esophageal surgeries were obtained from the NCD. In 2018, the data collection method for general thoracic and esophageal surgeries had been modified from self-reports using questionnaire sheets following each institution belonging to the JATS to an automatic package downloaded from the NCD in Japan.

The data collection related to cardiovascular surgery (initially self-reported using questionnaire sheets in each participating institution up to 2014) changed to downloading an automatic package from the Japanese Cardiovascular Surgery Database (JCVSD), which is a cardiovascular subsection of the NCD in 2015.

## Final report: 2024

### (A) Cardiovascular surgery

We are extremely pleased with the cooperation of our colleagues (members) in completing the cardiovascular surgery survey, which has undoubtedly improved the quality of this annual report. We are truly grateful for the significant efforts made by all participants within each participating institution in completing the JCVSD/NCD.

**Fig. 1:**
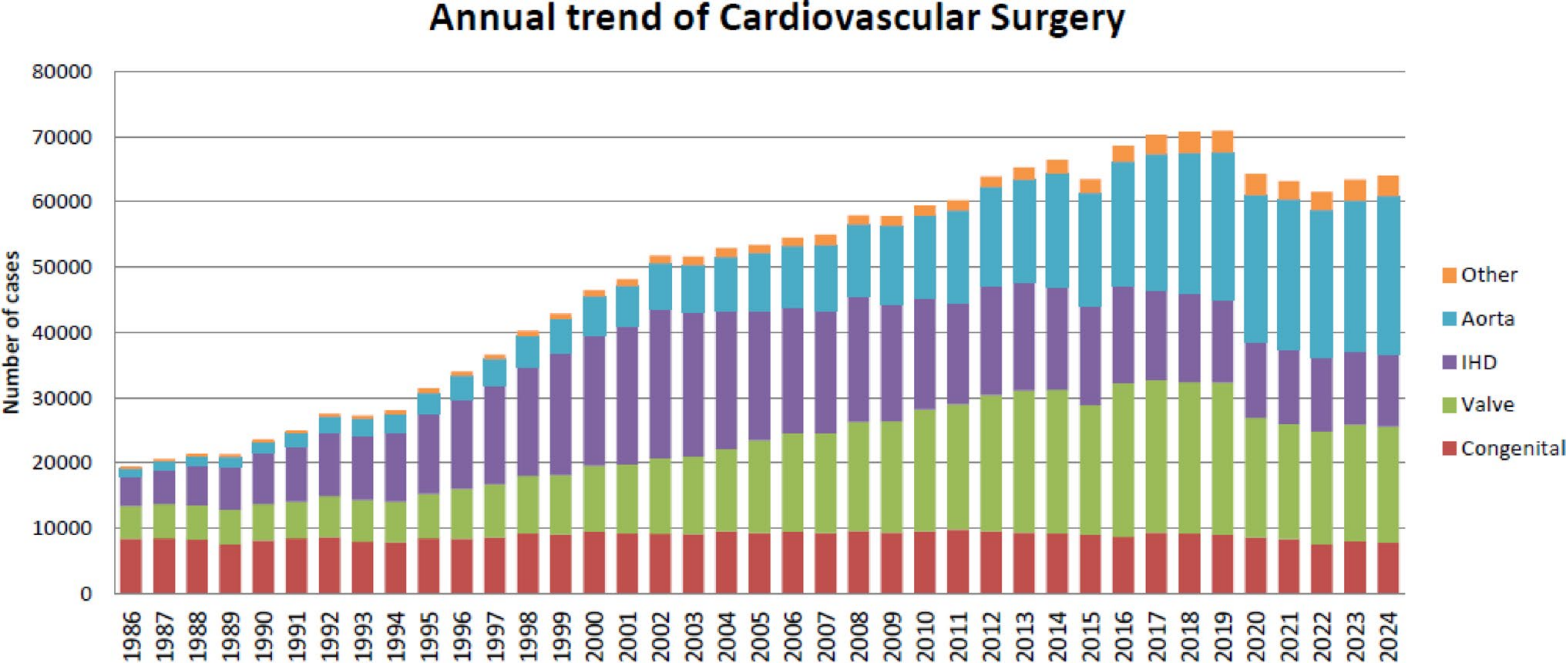
Cardiovascular surgery. IHD, ischemic heart disease

Figure 1 illustrates the development of cardiovascular surgery in Japan over the past 35 years. Aortic surgery includes only surgeries for aortic dissection, thoracic and thoracoabdominal aortic aneurysms. Extra-anatomic bypass surgery for thoracic aneurysm and pacemaker implantation have been excluded from the survey since 2015. Ventricular assist device (VAD) implantations had not been included in the total number of surgical procedures but we have decided to count the number of VAD implantation from previous Annual Report [7]. VAD implantations since 2016 were added to Fig. 1. A total of 64,047 cardiovascular surgeries, including 156 VAD implantations and 111 heart transplants, had been performed in 2024, with a 1.0% increase compared to that in 2023 (n = 63,427). Following on from 2020, a decline in the number of cases has been observed for the third consecutive year. In 2023, the downward trend finally came to a halt and started to increase. As the issues related to COVID-19 are being resolved, a gradual recovery in the number of surgeries is expected in the future.

Compared to data for 2023 [7] and 2014 [8], data for 2024 showed 2.7% (7866 vs. 8084) and 15.1% fewer surgeries for congenital heart disease, 0.2% (17,777 vs. 17,805) and 19.0% fewer surgeries for valvular heart disease, 2.3% (10,969 vs. 11,227) and 29.8% fewer surgeries for ischemic heart procedures, and 5.3% (24,333 vs. 23,104) and 39.1% more surgeries for thoracic aorta, respectively. Data for individual categories are summarized in Tables 1, 2, 3, 4, 5, 6.

**Table 1:** Congenital (total; 7866)

(1) CPB ( +) (total; 6022)																																			
	Neonate							Infant							1–17 years							≥ 18 years							Total						
	Cases	30-day mortality				Hospital mortality		Cases	30-day mortality				Hospital mortality		Cases	30-day mortality				Hospital mortality		Cases	30-day mortality				Hospital mortality		Cases	30-day mortality				Hospital mortality	
		Hospital		After discharge					Hospital		After discharge					Hospital		After discharge					Hospital		After discharge					Hospital		After discharge			
PDA	4	0		0		0		2	0		0		0		0	0		0		0		14	0		0		0		20	0		0		0	
Coarctation (simple)	12	0		0		0		7	0		0		0		11	0		0		0		9	0		0		0		39	0		0		0	
+ VSD	41	2	(4.9)	0		3	(7.3)	65	0		0		1	(1.5)	16	0		0		0		2	0		0		0		124	2	(1.6)	0		4	(3.2)
+ DORV	0	0		0		0		5	0		0		0		2	0		0		0		0	0		0		0		7	0		0		0	
+ AVSD	3	0		0		0		1	0		0		0		1	0		0		0		0	0		0		0		5	0		0		0	
+ TGA	0	0		0		0		0	0		0		0		0	0		0		0		0	0		0		0		0	0		0		0	
+ SV	0	0		0		0		2	0		0		0		2	0		0		0		0	0		0		0		4	0		0		0	
+ Others	5	0		0		0		9	0		0		1	(11.1)	3	0		0		0		3	0		0		0		20	0		0		1	(5.0)
Interrupt. of Ao (simple)	1	0		0		0		0	0		0		0		0	0		0		0		0	0		0		0		1	0		0		0	
+ VSD	16	1	(6.3)	0		2	(12.5)	26	1	(3.8)	0		2	(7.7)	10	0		0		0		0	0		0		0		52	2	(3.8)	0		4	(7.7)
+ DORV	0	0		0		0		0	0		0		0		0	0		0		0		0	0		0		0		0	0		0		0	
+ Truncus	4	0		0		0		7	0		0		0		1	0		0		0		0	0		0		0		12	0		0		0	
+ TGA	0	0		0		0		0	0		0		0		0	0		0		0		0	0		0		0		0	0		0		0	
+ Others	1	0		0		0		3	0		0		0		1	0		0		0		0	0		0		0		5	0		0		0	
Vascular ring	0	0		0		0		2	0		0		0		1	0		0		0		1	0		0		0		4	0		0		0	
PS	1	0		0		0		18	0		0		1	(5.6)	75	0		0		0		29	0		0		0		123	0		0		1	(0.8)
PA・IVS or Critical PS	6	1	(16.7)	0		1	(16.7)	52	1	(1.9)	0		2	(3.8)	43	0		0		0		4	0		0		0		105	2	(1.9)	0		3	(2.9)
TAPVR	80	8	(10.0)	0		13	(16.3)	40	1	(2.5)	0		2	(5.0)	17	0		0		0		0	0		0		0		137	9	(6.6)	0		15	(10.9)
PAPVR ± ASD	1	0		0		0		4	0		0		0		53	0		0		0		10	0		0		0		68	0		0		0	
ASD	3	1	(33.3)	0		1	(33.3)	30	0		1	(3.3)	0		383	0		0		0		796	10	(1.3)	0		10	(1.3)	1,212	11	(0.9)	1	(0.1)	11	(0.9)
Cor triatriatum	2	0		0		0		4	0		0		0		5	0		0		0		2	0		0		0		13	0		0		0	
AVSD (partial)	0	0		0		0		7	0		0		0		34	0		0		0		6	0		0		0		47	0		0		0	
AVSD (complete)	3	0		0		1	(33.3)	70	1	(1.4)	0		1	(1.4)	67	1	(1.5)	0		2	(3.0)	6	0		0		0		146	2	(1.4)	0		4	(2.7)
+ TOF or DORV	2	0		0		0		11	0		0		0		12	0		0		0		2	0		0		0		27	0		0		0	
+ Others	0	0		0		0		0	0		0		0		0	0		0		0		0	0		0		0		0	0		0		0	
VSD (subarterial)	0	0		0		0		85	0		0		0		126	0		0		0		9	0		0		0		220	0		0		0	
VSD (perimemb./muscular)	4	0		0		0		532	0		0		1	(0.2)	297	0		0		0		24	0		0		0		857	0		0		1	(0.1)
VSD (Type Unknown)	0	0		0		0		0	0		0		0		0	0		0		0		138	2	(1.4)	0		2	(1.4)	138	2	(1.4)	0		2	(1.4)
VSD + PS	0	0		0		0		16	0		0		0		13	0		0		0		3	0		0		0		32	0		0		0	
DCRV ± VSD	0	0		0		0		9	0		0		0		21	0		0		0		10	0		0		0		40	0		0		0	
Aneurysm of sinus of Valsalva	0	0		0		0		0	0		0		0		1	0		0		0		2	0		0		0		3	0		0		0	
TOF	16	1	(6.3)	0		2	(12.5)	137	1	(0.7)	0		2	(1.5)	143	0		0		2	(1.4)	60	0		0		1	(1.7)	356	2	(0.6)	0		7	(2.0)
PA + VSD	11	0		0		0		66	2	(3.0)	0		2	(3.0)	109	0		0		0		12	0		0		0		198	2	(1.0)	0		2	(1.0)
DORV	18	1	(5.6)	0		1	(5.6)	135	1	(0.7)	0		4	(3.0)	131	0		0		2	(1.5)	11	0		0		0		295	2	(0.7)	0		7	(2.4)
TGA (simple)	74	1	(1.4)	0		5	(6.8)	6	0		0		1	(16.7)	5	0		0		0		6	0		0		0		91	1	(1.1)	0		6	(6.6)
+ VSD	25	2	(8.0)	0		2	(8.0)	7	0		0		0		12	0		0		0		3	0		0		0		47	2	(4.3)	0		2	(4.3)
VSD + PS	1	0		0		0		0	0		0		0		0	0		0		0		0	0		0		0		1	0		0		0	
Corrected TGA	5	0		0		0		17	0		0		0		33	0		0		0		10	0		0		0		65	0		0		0	
Truncus arteriosus	5	0		0		0		20	0		0		2	(10.0)	25	0		0		0		3	0		0		0		53	0		0		2	(3.8)
SV	25	2	(8.0)	0		5	(20.0)	170	2	(1.2)	0		5	(2.9)	135	0		0		1	(0.7)	15	1	(6.7)	0		1	(6.7)	345	5	(1.4)	0		12	(3.5)
TA	4	0		0		0		24	0		0		0		38	0		0		0		7	0		0		0		73	0		0		0	
HLHS	16	2	(12.5)	0		3	(18.8)	72	2	(2.8)	0		3	(4.2)	57	0		0		1	(1.8)	2	0		0		0		147	4	(2.7)	0		7	(4.8)
Aortic valve lesion	7	1	(14.3)	0		2	(28.6)	16	0		0		0		80	1	(1.3)	0		2	(2.5)	38	0		0		0		141	2	(1.4)	0		4	(2.8)
Mitral valve lesion	1	0		0		0		19	3	(15.8)	0		3	(15.8)	58	0		0		0		23	0		0		0		101	3	(3.0)	0		3	(3.0)
Ebstein	1	0		0		0		11	1	(9.1)	0		2	(18.2)	24	1	(4.2)	0		2	(8.3)	10	0		0		0		46	2	(4.3)	0		4	(8.7)
Coronary disease	0	0		0		0		12	0		0		0		24	0		0		0		6	0		0		0		42	0		0		0	
Others	11	0		0		1	(9.1)	20	0		0		1	(5.0)	42	1	(2.4)	0		1	(2.4)	222	4	(1.8)	0		4	(1.8)	295	5	(1.7)	0		7	(2.4)
Conduit failure	0	0		0		0		0	0		0		0		30	0		0		0		7	0		0		0		37	0		0		0	
Redo (excluding conduit failure)	2	0		0		0		34	3	(8.8)	0		3	(8.8)	103	0		0		2	(1.9)	89	0		0		0		228	3	(1.3)	0		5	(2.2)
Total	411	23	(5.6)	0		42	(10.2)	1773	19	(1.1)	1	(0.06)	39	(2.2)	2244	4	(0.2)	0		15	(0.7)	1594	17	(1.1)	0		18	(1.1)	6022	63	(1.0)	1	(0.0)	114	(1.9)
(), % mortality																																			
CPB, cardiopulmonary bypass; PDA, patent ductus arteriosus; VSD, ventricular septal defect; DORV, double outlet right ventricle; AVSD, atrioventricular septal defect; TGA, transposition of great arteries; SV, single ventricle; Interrupt. of Ao., interruption of aorta																																			
PS, pulmonary stenosis; PA-IVS, pulmonary atresia with intact ventricular septum; TAPVR, total anomalous pulmonary venous return; PAPVR, partial anomalous pulmonary venous return; ASD, atrial septal defect; TOF, tetralogy of Fallot; DCRV, double-chambered right ventricle; TA, tricuspid atresia; HLHS, hypoplastic left heart syndrome; RV-PA, right ventricle-pulmonary artery																																			

(2) CPB (–) (total; 1844)																																			
	Neonate							Infant							1–17 years							≥ 18 years							Total						
	Cases	30-day mortality				Hospital mortality		Cases	30-day mortality				Hospital mortality		Cases	30-day mortality				Hospital mortality		Cases	30-day mortality				Hospital mortality		Cases	30-day mortality				Hospital mortality	
		Hospital		After discharge					Hospital		After discharge					Hospital		After discharge					Hospital		After discharge					Hospital		After discharge			
PDA	247	3	(1.2)	0		11	(4.5)	89	1	(1.1)	0		1	(1.1)	10	0		0		0		2	0		0		0		348	4	(1.1)	0		12	(3.4)
Coarctation (simple)	11	0		0		0		7	0		0		0		4	0		0		0		1	0		0		0		23	0		0		0	
+ VSD	50	0		0		2	(4.0)	14	0		0		2	(14.3)	1	0		0		0		0	0		0		0		65	0		0		4	(6.2)
+ DORV	2	0		0		0		1	0		0		0		0	0		0		0		0	0		0		0		3	0		0		0	
+ AVSD	1	0		0		0		0	0		0		0		0	0		0		0		0	0		0		0		1	0		0		0	
+ TGA	0	0		0		0		0	0		0		0		0	0		0		0		0	0		0		0		0	0		0		0	
+ SV	0	0		0		0		1	0		0		0		0	0		0		0		0	0		0		0		1	0		0		0	
+ Others	1	0		0		0		4	1	(25.0)	0		1	(25.0)	0	0		0		0		0	0		0		0		5	1	(20.0)	0		1	(20.0)
Interrupt. of Ao (simple)	0	0		0		0		0	0		0		0		0	0		0		0		0	0		0		0		0	0		0		0	
+ VSD	29	2	(6.9)	0		2	(6.9)	5	0		0		1	(20.0)	2	0		0		0		0	0		0		0		36	2	(5.6)	0		3	(8.3)
+ DORV	0	0		0		0		0	0		0		0		0	0		0		0		0	0		0		0		0	0		0		0	
+ Truncus	7	0		0		0		2	0		0		0		1	0		0		0		0	0		0		0		10	0		0		0	
+ TGA	0	0		0		0		0	0		0		0		0	0		0		0		0	0		0		0		0	0		0		0	
+ Others	2	1	(50.0)	0		1	(50.0)	1	0		0		0		1	0		0		0		0	0		0		0		4	1	(25.0)	0		1	(25.0)
Vascular ring	6	0		0		0		13	0		0		0		20	0		0		0		0	0		0		0		39	0		0		0	
PS	0	0		0		0		6	0		0		0		5	0		0		0		0	0		0		0		11	0		0		0	
PA・IVS or Critical PS	14	2	(14.3)	0		2	(14.3)	22	0		0		0		3	0		0		0		2	0		0		0		41	2	(4.9)	0		2	(4.9)
TAPVR	24	3	(12.5)	0		3	(12.5)	6	0		0		0		6	0		0		0		0	0		0		0		36	3	(8.3)	0		3	(8.3)
PAPVR ± ASD	0	0		0		0		0	0		0		0		0	0		0		0		0	0		0		0		0	0		0		0	
ASD	1	0		0		0		1	0		0		0		5	0		0		0		1	0		0		0		8	0		0		0	
Cor triatriatum	0	0		0		0		0	0		0		0		0	0		0		0		0	0		0		0		0	0		0		0	
AVSD (partial)	1	0		0		0		1	0		0		0		2	0		0		0		2	0		0		0		6	0		0		0	
AVSD (complete)	58	1	(1.7)	0		1	(1.7)	57	0		0		1	(1.8)	8	0		0		0		0	0		0		0		123	1	(0.8)	0		2	(1.6)
+ TOF or DORV	0	0		0		0		4	0		0		0		1	0		0		0		0	0		0		0		5	0		0		0	
+ Others	0	0		0		0		0	0		0		0		0	0		0		0		0	0		0		0		0	0		0		0	
VSD (subarterial)	5	0		0		0		12	0		0		1	(8.3)	0	0		0		0		0	0		0		0		17	0		0		1	(5.9)
VSD (perimemb./muscular)	57	0		0		0		115	0		0		1	(0.9)	13	0		0		0		0	0		0		0		185	0		0		1	(0.5)
VSD (Type Unknown)	0	0		0		0		0			0		0		0	0		0		0		1	0		0		0		1	0		0		0	
VSD + PS	0	0		0		0		2	0		0		0		0	0		0		0		0	0		0		0		2	0		0		0	
DCRV ± VSD	0	0		0		0		0	0		0		0		0	0		0		0		0	0		0		0		0	0		0		0	
Aneurysm of sinus of Valsalva	0	0		0		0		0	0		0		0		0	0		0		0		0	0		0		0		0	0		0		0	
TOF	18	1	(5.6)	0		1	(5.6)	48	0		0		1	(2.1)	5	1	(20.0)	0		2	(40.0)	6	0		0		0		77	2	(2.6)	0		4	(5.2)
PA + VSD	7	0		0		0		44	1	(2.3)	0		2	(4.5)	13	0		0		0		0	0		0		0		64	1	(1.6)	0		2	(3.1)
DORV	51	1	(2.0)	0		1	(2.0)	61	1	(1.6)	0		3	(4.9)	9	0		0		0		3	0		0		0		124	2	(1.6)	0		4	(3.2)
TGA (simple)	18	0		0		0		8	1	(12.5)	0		1	(12.5)	0	0		0		0		2	0		0		0		28	1	(3.6)	0		1	(3.6)
+ VSD	9	2	(22.2)	0		2	(22.2)	5	0		0		0		2	0		0		0		0	0		0		0		16	2	(12.5)	0		2	(12.5)
VSD + PS	0	0		0		0		0	0		0		0		0	0		0		0		0	0		0		0		0	0		0		0	
Corrected TGA	3	0		0		0		12	0		0		0		8	0		0		0		1	0		0		0		24	0		0		0	
Truncus arteriosus	16	0		0		3	(18.8)	2	0		0		0		5	0		0		0		0	0		0		0		23	0		0		3	(13.0)
SV	51	2	(3.9)	0		4	(7.8)	55	3	(5.5)	0		4	(7.3)	28	1	(3.6)	0		1	(3.6)	5	0		0		0		139	6	(4.3)	0		9	(6.5)
TA	16	1	(6.3)	0		1	(6.3)	7	0		0		0		2	0		0		0		5	0		0		0		30	1	(3.3)	0		1	(3.3)
HLHS	63	2	(3.2)	0		4	(6.3)	36	1	(2.8)	0		1	(2.8)	13	0		0		0		0	0		0		0		112	3	(2.7)	0		5	(4.5)
Aortic valve lesion	3	1	(33.3)	0		2	(66.7)	2	0		0		0		4	2	(50.0)	0		2	(50.0)	1	0		0		0		10	3	(30.0)	0		4	(40.0)
Mitral valve lesion	0	0		0		0		6	3	(50.0)	0		3	(50.0)	1	0		0		0		0	0		0		0		7	3	(42.9)	0		3	(42.9)
Ebstein	1	0		0		0		6	1	(16.7)	0		1	(16.7)	0	0		0		0		0	0		0		0		7	1	(14.3)	0		1	(14.3)
Coronary disease	0	0		0		0		3	0		0		0		9	0		0		0		1	0		0		1	(100.0)	13	0		0		1	(7.7)
Others	12	1	(8.3)	0		1	(8.3)	7	1	(14.3)	0		1	(14.3)	8	1	(12.5)	0		1	(12.5)	5	0		0		1	(20.0)	32	3	(9.4)	0		4	(12.5)
Conduit failure	0	0		0		0		0	0		0		0		0	0		0		0		1	0		0		0		1	0		0		0	
Redo (excluding conduit failure)	11	0		0		0		60	3	(5.0)	0		4	(6.7)	74	4	(5.4)	0		5	(6.8)	22	0		0		1	(4.5)	167	7	(4.2)	0		10	(6.0)
Total	795	23	(2.9)	0		41	(5.2)	725	17	(2.3)	0		29	(4.0)	263	9	(3.4)	0		11	(4.2)	61	0		0		3	(4.9)	1,844	49	(2.7)	0		84	(4.6)
(), % mortality																																			
CPB, cardiopulmonary bypass; PDA, patent ductus arteriosus; VSD, ventricular septal defect; DORV, double outlet right ventricle; AVSD, atrioventricular septal defect; TGA, transposition of the great arteries; SV, single ventricle; Interrupt. of Ao., interruption of aorta																																			
PS, pulmonary stenosis; PA-IVS, pulmonary atresia with intact ventricular septum; TAPVR, total anomalous pulmonary venous return; PAPVR, partial anomalous pulmonary venous return; ASD, atrial septal defect; TOF, tetralogy of Fallot; DCRV, double-chambered right ventricle; TA, tricuspid atresia; HLHS, hypoplastic left heart syndrome; RV-PA, right ventricle-pulmonary artery																																			

(3)Main procedure																																				
		Neonate							Infant							1–17 years							≥ 18 years							Total						
		Cases	30-day mortality				Hospital mortality		Cases	30-day mortality				Hospital mortality		Cases	30-day mortality				Hospital mortality		Cases	30-day mortality				Hospital mortality		Cases	30-day mortality				Hospital mortality	
					After discharge					Hospital		After discharge					Hospital		After discharge					Hospital		After discharge					Hospital		After discharge			
1	SP Shunt	87	3	(3.4)	0		3	(3.4)	296	6	(2.0)	0		9	(3.0)	31	0		0		1	(3.2)	0	0		0		0		414	9	(2.2)	0		13	(3.1)
2	PAB	251	8	(3.2)	0		15	(6.0)	271	1	(0.4)	0		9	(3.3)	14	0		0		0		1	0		0		0		537	9	(1.7)	0		24	(4.5)
3	Bidirectional Glenn or hemi-Fontan ±α	0	0		0		0		193	0		0		0		59	0		0		0		1	0		0		0		253	0		0		0	
4	Damus-Kaye-Stansel operation	1	0		0		0		18	0		0		1	(5.6)	3	0		0		0		0	0		0		0		22	0		0		1	(4.5)
5	PA reconstruction/repair (including redo)	22	1	(4.5)	0		3	(13.6)	149	2	(1.3)	0		6	(4.0)	197	0		0		1	(0.5)	28	0		0		0		396	3	(0.8)	0		10	(2.5)
6	RVOT reconstruction/repair	4	0		0		0		203	0		0		1	(0.5)	282	0		0		1	(0.4)	50	0		0		0		539	0		0		2	(0.4)
7	Rastelli procedure	2	1	(50.0)	0		1	(50.0)	45	0		0		0		101	1	(1.0)	0		2	(2.0)	4	0		0		0		152	2	(1.3)	0		3	(2.0)
8	Arterial switch procedure	113	2	(1.8)	0		7	(6.2)	17	0		0		1	(5.9)	2	0		0		0		0	0		0		0		132	2	(1.5)	0		8	(6.1)
9	Atrial switch procedure	1	0		0		0		2	1	(50.0)	0		1	(50.0)	6	0		0		0		3	0		0		0		12	1	(8.3)	0		1	(8.3)
10	Double switch procedure	0	0		0		0		1	0		0		0		7	0		0		0		1	0		0		0		9	0		0		0	
11	Repair of anomalous origin of CA	0	0		0		0		4	0		0		0		4	0		0		0		1	0		0		0		9	0		0		0	
12	Closure of coronary AV fistula	0	0		0		0		3	0		0		0		6	0		0		1	(16.7)	1	0		0		0		10	0		0		1	(10.0)
13	Fontan/TCPC	0	0		0		0		0	0		0		0		228	0		0		1	(0.4)	27	1	(3.7)	0		1	(3.7)	255	1	(0.4)	0		2	(0.8)
14	Norwood procedure	12	2	(16.7)	0		2	(16.7)	49	1	(2.0)	0		2	(4.1)	2	0		0		0		0	0		0		0		63	3	(4.8)	0		4	(6.3)
15	Ventricular septation	0	0		0		0		0	0		0		0		1	0		0		0		0	0		0		0		1	0		0		0	
16	Left side AV valve repair (including Redo)	2	0		0		1	(50.0)	33	2	(6.1)	0		2	(6.1)	72	0		0		0		22	0		0		0		129	2	(1.6)	0		3	(2.3)
17	Left side AV valve replace (including Redo)	0	0		0		0		8	1	(12.5)	0		1	(12.5)	39	1	(2.6)	0		1	(2.6)	22	0		0		1	(4.5)	69	2	(2.9)	0		3	(4.3)
18	Right side AV valve repair (including Redo)	4	0		0		0		95	1	(1.1)	0		3	(3.2)	94	0		0		1	(1.1)	57	0		0		0		250	1	(0.4)	0		4	(1.6)
19	Right side AV valve replace (including Redo)	0	0		0		0		2	1	(50.0)	0		1	(50.0)	7	0		0		0		26	0		0		1	(3.8)	35	1	(2.9)	0		2	(5.7)
20	Common AV valve repair (including Redo)	4	0		0		2	(50.0)	20	1	(5.0)	0		4	(20.0)	16	0		0		0		1	0		0		0		41	1	(2.4)	0		6	(14.6)
21	Common AV valve replace (including Redo)	0	0		0		0		2	1	(50.0)	0		1	(50.0)	16	0		0		0		3	0		0		0		21	1	(4.8)	0		1	(4.8)
22	Repair of supra-aortic stenosis	0	0		0		0		5	0		0		0		12	0		0		0		3	0		0		0		20	0		0		0	
23	Repair of subaortic stenosis (including Redo)	1	0		0		0		7	0		0		0		27	0		0		0		3	0		0		0		38	0		0		0	
24	Aortic valve plasty ± VSD Closure	5	0		0		1	(20.0)	16	1	(6.3)	0		1	(6.3)	31	0		0		0		3	0		0		0		55	1	(1.8)	0		2	(3.6)
25	Aortic valve replacement	1	0		0		0		0	0		0		0		22	0		0		1	(4.5)	37	0		0		1	(2.7)	60	0		0		2	(3.3)
26	AVR with annular enlargement	0	0		0		0		0	0		0		0		8	1	(12.5)	0		1	(12.5)	5	0		0		0		13	1	(7.7)	0		1	(7.7)
27	Aortic root Replace (except Ross)	0	0		0		0		0	0		0		0		6	0		0		0		18	0		0		0		24	0		0		0	
28	Ross procedure	0	0		0		0		5	0		0		0		18	0		0		0									23	0		0		0	
29	Bilateral pulmonary artery banding	181	3	(1.7)	0		17	(9.4)	19	1	(5.3)	0		2	(10.5)	0	0		0		0		0	0		0		0		200	4	(2.0)	0		19	(9.5)
Total		691	20	(2.9)	0		52	(7.5)	1,463	20	(1.4)	0		45	(3.1)	1,311	3	(0.2)	0		11	(0.8)	317	1	(0.3)	0		4	(1.3)	3,782	44	(1.2)	0		112	(3.0)
(), % mortality																																				
SP, systemic-pulmonary; PAB pulmonary artery banding; PA, pulmonary artery; RVOT, right ventricular outflow tract; CA, coronary artery; AV fistula, arteriovenous fistula; TCPC, total cavopulmonary connection; AV valve, atrioventricular valve; VSD, ventricular septal defect; AVR, aortic valve replacement

**Table 2:** Acquired (total, (1) + (2) + (4) + (5) + (6) + (7) + isolated operations for arrhythmia in (3); 31,404

(1)Valvelar heart disease (total; 17,777)																	
	Valve	Cases	Operation					30-Day mortality				Hospital mortality		Redo			
			Mechanical	Bioprosthesis	Repair	Unknown	With CABG	Hospital		After discharge				Cases	30-Day mortality		Hospital mortality
								Replace	Repair	Replace	Repair	Replace	Repair		Hosipital	After discharge	
Isolated	A	7711	963	6602	104	42	1640	114 (1.5)	3 (2.9)	3 (0.04)	0	191 (2.5)	4 (3.8)	624	26 (4.2)	0	37 (5.9)
	M	5393	340	981	4036	36	525	48 (3.6)	33 (0.8)	1 (0.08)	3 (0.07)	91 (6.9)	54 (1.3)	560	18 (3.2)	1(0.2)	32 (5.7)
	T	206	2	49	152	3	27	4 (7.8)	5 (3.3)	0	0	4(7.8)	7 (4.6)	59	0	0	2 (3.4)
	P	16	1	15	0	0	1	0		0		0		11	0	0	0
A + M		991					159	35 (3.5)	0	1 (0.1)	0	61 (6.2)	0	166	7 (4.2)	1(0.6)	15 (9.0)
	A		152	810	28	1											
	M		118	386	475	12											
A + T		293					43	8 (2.7)		0		12 (4.1)		47	1(2.1)	0	3 (6.4)
	A		21	262	9	1											
	T		0	0	284	9											
M + T		2515					168	36 (1.4)		0		63 (2.5)		275	5 (1.8)	1(0.4)	12 (4.4)
	M		172	739	1589	15											
	T		0	17	2483	15											
A + M + T		583					53	17 (2.9)		0		30 (5.1)		98	3 (3.1)	0	6 (6.1)
	A		61	508	14	0											
	M		46	246	287	4											
	T		2	9	571	1											
Others		69					6	1 (1.4)		0		3 (4.3)		20	0	0	1(5.0)
Total		17,777					2,622	263 (1.5)		5 (0.03)		455 (2.6)		1860	60 (3.2)	3(0.2)	108 (5.8)
TAVR								Cases								30-day mortality	
								16,261									

(2) Ischemic heart disease (total, (A) + (B) ; 10,969)																																	
(A) Isolated CABG (total; (a)+(b); 9,840)																																	
(a-1) On-pump arrest CABG (total;2,143)																																	
	Primary, elective							Primary, emergent							Redo, elective							Redo, emergent							Artery only	Artery +SVG	SVG only	Others	Unclear
	Cases	30 day mortality				Hospital mortality		Cases	30 day mortality				Hospital mortality		Cases	30 day mortality				Hospital mortality		Cases	30 day mortality				Hospital mortality						
		Hospital		After discharge					Hospital		After discharge					Hospital		After discharge					Hospital		After discharge								
1VD	52	2	(3.8)	0		2	(3.8)	9	1	(11.1)	0		2	(22.2)	2	0		0		0		1	0		0		0		25	22	15	1	1
2VD	263	2	(0.8)	0		4	(1.5)	20	2	(10.0)	0		2	(10.0)	3	1	(33.3)	0		1	(33.3)	1	0		0		0		36	233	17	1	0
3VD	790	11	(1.4)	1	(0.13)	18	(2.3)	68	6	(8.8)	0		7	(10.3)	3	0		0		0		0	0		0		0		48	785	18	9	1
LMT	717	9	(1.3)	0		14	(2.0)	150	15	(10.0)	1	(0.7)	15	(10.0)	7	2	(28.6)	0		2	(28.6)	0	0		0		0		58	783	29	4	0
No info	45	2	(4.4)	0		5	(11.1)	11	1	(9.1)	0		1	(9.1)	1	0		0		0		0	0		0		0		7	30	18	1	1
Total	1867	26	(1.4)	1	(0.05)	43	(2.3)	258	25	(9.7)	1	(0.4)	27	(10.5)	16	3	(18.8)	0		3	(18.8)	2	0		0		0		174	1853	97	16	3
Kawasaki	8	0		0		0		0	0		0		0		0	0		0		0		0	0		0		0		5	2	1	0	0
On dialysis	201	11	(5.5)	0		18	(9.0)	32	8	(25.0)	1	(3.1)	9	(28.1)	3	0		0		0		0	0		0		0		14	210	10	2	0
(), % mortality																																	
CABG, coronary artery bypass grafting; 1VD, one-vessel disease; 2VD two-vessel disease; 3VD, three-vessel disease; LMT, left main trunk; SVG, saphenous vein graft; LMT includes LMT alone or LMT with other branch diseases.																																	

(a-2) On-pump beating CABG (total;2,085)																																	
	Primary, elective							Primary, emergent							Redo, elective							Redo, emergent							Artery only	Artery +SVG	SVG only	Others	Unclear
	Cases	30 day mortality				Hospital mortality		Cases	30 day mortality				Hospital mortality		Cases	30 day mortality				Hospital mortality		Cases	30 day mortality				Hospital mortality						
		Hospital		After discharge					hospital		After discharge					hospital		After discharge					Hospital		After discharge								
1VD	29	0		0		0		12	2	(16.7)	0		2	(16.7)	1	0		0		0		1	0		0		0		15	13	15	0	0
2VD	220	5	(2.3)	0		8	(3.6)	30	4	(13.3)	0		5	(16.7)	6	1	(16.7)	0		1	(16.7)	0	0		0		0		52	184	17	3	0
3VD	769	16	(2.1)	1	(0.1)	23	(3.0)	112	8	(7.1)	0		14	(12.5)	4	0		0		0		0	0		0		0		83	764	27	10	1
LMT	666	13	(2.0)	0		25	(3.8)	193	11	(5.7)	1	(0.5)	23	(11.9)	7	1	(14.3)	0		1	(14.3)	2	0		0		0		137	695	30	4	2
no info	18	0		0		0		9	1	(11.1)	0		1	(11.1)	2	0		0		0		4	3	(75.0)	0		3	(75.0)	10	13	10	0	0
Total	1702	34	(2.0)	1	(0.06)	56	(3.3)	356	26	(7.3)	1		45	(12.6)	20	2	(10.0)	0		2	(10.0)	7	3	(42.9)	0		3	(42.9)	297	1669	99	17	3
kawasaki	2	0		0		0		0	0		0		0		0	0		0		0		0	0		0		0		1	1	0	0	0
on dialysis	246	12	(4.9)	1	(0.4)	19	(7.7)	37	7	(18.9)	1	(2.7)	10	(27.0)	3	1	(33.3)	0		1	(33.3)	1	1	(100.0)	0		1	(100.0)	18	249	16	3	1
(), % mortality																																	
CABG, coronary artery bypass grafting; 1VD, one-vessel disease; 2VD two-vessel disease; 3VD, three-vessel disease; LMT, left main trunk; SVG, saphenous vein graft; LMT includes LMT alone or LMT with other branch diseases.																																	

(b) Off-pump CABG (total;5,612)																																	
(Including cases of planned off-pump CABG in which, during surgery, the change is made to an on-pump CABG or on-pump beating-heart procedure）																																	
	Primary, elective							Primary, emergent							Redo, elective							Redo, emergent							Artery only	Artery +SVG	SVG only	Others	Unclear
	Cases	30 day mortality				Hospital mortality		Cases	30 day mortality				Hospital mortality		Cases	30 day mortality				Hospital mortality		Cases	30 day mortality				Hospital mortality						
		Hospital		After discharge					Hospital		After discharge					Hospital		After discharge					Hospital		After discharge								
1VD	329	1	(0.3)	0		2	(0.6)	30	7	(23.3)	0		9	(30.0)	5	0		0		0		1	0		0		0		292	47	25	1	0
2VD	817	8	(1.0)	0		11	(1.3)	70	1	(1.4)	0		2	(2.9)	5	0		0		0		2	0		0		0		316	548	26	3	1
3VD	2003	25	(1.2)	2	(0.1)	34	(1.7)	160	10	(6.3)	0		17	(10.6)	4	1	(25.0)	0		1	(25.0)	2	1	(50.0)	0		1	(50.0)	454	1681	29	5	0
LMT	1742	13	(0.7)	0		27	(1.5)	292	8	(2.7)	0		15	(5.1)	14	0		0		0		3	0		0		1	(33.3)	607	1393	41	10	0
no info	105	2	(1.9)	0		4	(3.8)	23	1	(4.3)	0		5	(21.7)	4	0		0		0		1	0		0		0		58	64	9	2	0
Total	4996	49	(1.0)	2	(0.0)	78	(1.6)	575	27	(4.7)	0		48	(8.3)	32	1	(3.1)	0		1	(3.1)	9	1	(11.1)	0		2	(22.2)	1727	3733	130	21	1
kawasaki	22	0		0		0		0	0		0		0		1	0		0		0		0	0		0		0		16	7	0	0	0
on dialysis	475	13	(2.7)	0		25	(5.3)	46	5	(10.9)	0		10	(21.7)	5	0		0		0		0	0		0		0		114	397	12	3	0
(), % mortality																																	
CABG, coronary artery bypass grafting; 1VD, one-vessel disease; 2VD two-vessel disease; 3VD, three-vessel disease; LMT, left main trunk; SVG, saphenous vein graft																																	
LMT includes LMT alone or LMT with other branch diseases.																																	

(c) Cases of conversion, during surgery, from off-pump CABG to on-pump CABG or on- pump beating-heart CABG (these cases are also included in category (b))																												
	Primary, elective							Primary, emergent							Redo, elective							Redo, emergent						
	Cases	30 day mortality				Hospital mortality		Cases	30 day mortality				Hospital mortality		Cases	30 day mortality				Hospital mortality		Cases	30 day mortality				Hospital mortality	
		Hospital		After discharge					Hospital		After discharge					Hospital		After discharge					Hospital		After discharge			
Converted to arrest	17	1	(5.9)	0		2	(11.8)	2	0		0		0		0	0		0		0		1	0		0		0	
Converted to beating	87	9	(10.3)	0		13	(14.9)	19	1	(5.3)	0		2	(10.5)	1	0		0		0		0	0		0		0	
Total	104	10	(9.6)	0		15	(14.4)	21	1	(4.8)	0		2	(9.5)	1	0		0		0		1	0		0		0	
On dialysis	18	2	(11.1)	0		3	(16.7)	2	0		0		0		0	0		0		0		0	0		0		0	
(), % mortality																												
CABG, coronary artery bypass grafting																												

(B) Operation for complications of MI (total; 1,129)																	
	Chronic							Acute							Concomitant operation		
	Cases	30-day mortality				Hospital mortality		Cases	30-day mortality				Hospital mortality				
		Hospital		After discharge					Hospital		After discharge				CABG	MVP	MVR
Infarctectomy or Aneurysmectomy	83	1	(1.2)	0		1	(1.2)	23	4	(17.4)	0		6	(26.1)	54	15	3
VSP closure	98	10	(10.2)	0		17	(17.3)	279	61	(21.9)	0		89	(31.9)	100	6	3
Cardiac rupture	32	7	(21.9)	0		8	(25.0)	236	69	(29.2)	0		84	(35.6)	30	0	3
Mitral regurgitation																	
1) Papillary muscle rupture	18	1	(5.6)	0		2	(11.1)	55	5	(9.1)	0		8	(14.5)	22	7	66
2) Ischemic	94	10	(10.6)	0		15	(16.0)	39	10	(25.6)	0		12	(30.8)	92	72	61
Others	81	2	(2.5)	0		5	(6.2)	91	22	(24.2)	0		29	(31.9)	68	6	0
Total	406	31	(7.6)	0		48	(11.8)	723	171	(23.7)	0		228	(31.5)	366	106	136
(), % mortality																	
MI, myocardial infarction; CABG,coronary artery bypass grafting; MVP, mitral valve repair; MVR, mitral valve replacement; VSP, ventricular septal perforation																	
Acute, within 2 weeks from the onset of myocardial infarction																	

(3) Operation for arrhythmia (total;6,507 )														
	Cases	30-day mortality				Hospital mortality		Concomitant operation						
								Isolated	Congenital	Valve	IHD	Others	Multiple combination	
		Hospital		After discharge									2 categories	3 categories
Maze	3,038	53	(1.7)	2	(0.07)	86	(2.8)	342	137	2,401	408	295	485	46
For WPW	0	0		0		0		0	0	0	0	0	0	0
For ventricular tachyarrhythmia	22	1	(4.5)	0		2	(9.1)	3	1	11	6	2	3	0
Others	3,447	72	(2.1)	2	(0.06)	122	(3.5)	310	165	2,576	637	401	591	49
Total	6,507	126	(1.9)	4	(0.06)	210	(3.2)	655	303	4,988	1051	698	1,079	95
(), % mortality														
WPW, Wolff-Parkinson-White syndrome; IHD, ischemic heart disease														
Except for 170 isolated cases, all remaining 5,164 cases are doubly allocated, one for this subgroup and the other for the subgroup corresponding to the concomitant operations.														

(4) Operation for constrictive pericarditis (total; 99)														
	CPB ( +)							CPB (–)						
	Cases	30-day mortality				Hospital mortality		Cases	30-day mortality				Hospital mortality	
		Hospital		After discharge					Hospital		After discharge			
Total	99	6	(6.1)	0		17	(17.2)	75	5	(6.7)	0		8	(10.7)
(), % mortality														
CPB, cardiopulmonary bypass														

(5) Cardiac tumor (total; 650)											
	Cases	30-day mortality				Hospital mortality		Concomitant operation			
		Hospital		After discharge				AVR	MVR	CABG	others
Benign tumor	577	3	(0.5)	0		6	(1.0)	36	18	43	113
(Cardiac myxoma)	399	2	(0.5)	0		3	(0.8)	12	6	22	67
Malignant tumor	73	5	(6.8)	0		5	(6.8)	1	2	1	21
(Primary)	42	2	(4.8)	0		2	(4.8)	1	2	1	13
(), % mortality											
AVR, aortic valve replacement; MVR, mitral valve replacement; CABG, coronary artery bypass grafting											

(6) HOCM and DCM (total; 218)											
	Cases	30-day mortality				Hospital mortality		Concomitant operation			
		Hospital		After discharge				AVR	MVR	MVP	CABG
Myectomy	128	2	(1.6)	0		2	(1.6)	50	17	20	12
Myotomy	5	0		0		0		2	1	1	0
No-resection	80	3	(3.8)	0		5	(6.3)	18	34	46	5
Volume reduction surgery of the left ventricle	5	0		0		1	(20.0)	1	1	0	0
Total	218	5	(2.3)	0		8	(3.7)	71	53	67	17
(), % mortality											
HOCM, hypertrophic obstructive cardiomyopathy; DCM, dilated cardiomyopathy; AVR, aortic valve replacement; MVR, mitral valve replacement; MVP, mitral valve repair, CABG, coronary artery bypass grafting											

(7) Other open-heart operation (total; 1,036)							
	Cases	30-day mortality				Hospital mortality	
		Hospital		After discharge			
Open-heart operation	461	58	(12.6)	0		71	(15.4)
Non-open-heart operation	575	80	(13.9)	1	(0.2)	111	(19.3)
Total	1036	138	(13.3)	1	(0.1)	182	(17.6)
(), % mortality

**Table 3:** Thoracic aorta (total; 24,333)

(1) Dissection (total; 12,195)																																		
Stanford type	Acute														Chronic														Concomitant operation					
	A							B							A							B												
Replaced site	Cases	30-day mortality				Hospital mortality		Cases	30-day mortality				Hospital mortality		Cases	30-day mortality				Hospital mortality		Cases	30-day mortality				Hospital mortality		AVP	AVR	MVP	MVR	CABG	Others
		Hospital		After discharge					Hospital		After discharge					Hospital		After discharge					Hospital		After discharge									
Ascending Ao	1928	147	(7.6)	0		188	(9.8)	2	1	(50.0)	0		1	(50.0)	187	3	(1.6)	0		4	(2.1)	3	0		0		0		43	130	12	10	102	28
Aortic Root	234	31	(13.2)	0		38	(16.2)	1	1	(100.0)	0		1	(100.0)	66	1	(1.5)	0		3	(4.5)	4	1	(25.0)	0		1	(25.0)	49	188	4	1	54	5
Arch	2157	138	(6.4)	2	(0.09)	181	(8.4)	24	1	(4.2)	0		2	(8.3)	415	7	(1.7)	0		12	(2.9)	154	3	(1.9)	0		6	(3.9)	79	135	10	1	111	22
Aortic root + asc. Ao. + Arch	186	22	(11.8)	0		30	(16.1)	1	0		0		0		43	2	(4.7)	0		2	(4.7)	4	0		0		0		34	145	6	0	52	3
Descending Ao	35	3	(8.6)	0		5	(14.3)	31	2	(6.5)	0		3	(9.7)	73	0		0		0		189	6	(3.2)	0		9	(4.8)	4	2	0	0	2	0
Thoracoabdominal	0	0		0		0		13	0		0		0		52	6	(11.5)	0		7	(13.5)	174	10	(5.7)	0		13	(7.5)	0	0	0	0	0	0
Simple TEVAR	130	8	(6.2)	0		9	(6.9)	497	42	(8.5)	0		51	(10.3)	292	3	(1.0)	0		3	(1.0)	1220	14	(1.1)	1	(0.1)	20	(1.6)	0	2	0	0	0	2
Open SG with BR	1894	173	(9.1)	0		203	(10.7)	96	5	(5.2)	0		6	(6.3)	239	5	(2.1)	0		11	(4.6)	293	2	(0.7)	0		6	(2.0)	83	193	9	2	152	12
Open SG without BR	552	36	(6.5)	0		53	(9.6)	30	1	(3.3)	0		1	(3.3)	83	1	(1.2)	0		3	(3.6)	79	1	(1.3)	0		2	(2.5)	21	58	3	4	32	4
Arch TEVAR with BR	25	5	(20.0)	0		5	(20.0)	128	6	(4.7)	0		7	(5.5)	91	0		0		1	(1.1)	434	8	(1.8)	1	(0.2)	8	(1.8)	0	0	0	0	0	1
Thoracoabdominal TEVAR with BR	0	0		0		0		5	0		0		0		7	0		0		0		36	1	(2.8)	0		1	(2.8)	0	0	0	0	0	0
Other	17	6	(35.3)	0		8	(47.1)	17	2	(11.8)	0		2	(11.8)	13	0		0		0		41	1	(2.4)	0		2	(4.9)	0	1	0	0	2	1
Total	7158	569	(7.9)	2	(0.03)	720	(10.1)	845	61	(7.2)	0		74	(8.8)	1561	28	(1.8)	0		46	(2.9)	2631	47	(1.8)	2	(0.1)	68	(2.6)	313	854	44	18	507	78
(), % mortality																																		
Ao, aorta; AVP, aortic valve repair; AVR aortic valve replacement; MVP, mitral valve repair; MVR, mitral valve replacement; CABG. coronary artery bypass grafting; TEVAR, thoracic endovascular aortic (aneurysm) repair																																		
Acute, within 2 weeks from the onset																																		

(2) Non-dissection (total; 12,138)																				
Replaced site	Unruptured							Ruptured							Concomitant operation					
	Cases	30-day mortality				Hospital mortality		Cases	30-day mortality				Hospital mortality		AVP	AVR	MVP	MVR	CABG	Others
		Hospital		After discharge					Hospital		After discharge									
Ascending Ao	1294	28	(2.2)	1	(0.1)	42	(3.2)	64	6	(9.4)	0		8	(12.5)	28	903	66	34	148	90
Aortic Root	1205	31	(2.6)	0		41	(3.4)	63	6	(9.5)	0		10	(15.9)	278	880	65	32	168	73
Arch	2101	41	(2.0)	1	(0.0)	71	(3.4)	101	10	(9.9)	0		17	(16.8)	31	640	37	20	209	62
Aortic root + asc. Ao. + Arch	298	9	(3.0)	0		13	(4.4)	9	2	(22.2)	0		2	(22.2)	62	223	6	3	35	10
Descending Ao	286	11	(3.8)	1	(0.3)	16	(5.6)	35	8	(22.9)	1	(2.9)	9	(25.7)	0	11	0	1	8	1
Thoracoabdominal	346	21	(6.1)	0		33	(9.5)	33	6	(18.2)	0		8	(24.2)	0	0	0	0	0	0
Simple TEVAR	2459	40	(1.6)	6	(0.2)	65	(2.6)	339	41	(12.1)	3	(0.9)	60	(17.7)	0	0	0	0	1	3
Open SG with BR	1520	34	(2.2)	2	(0.1)	64	(4.2)	73	9	(12.3)	0		17	(23.3)	20	181	9	1	189	25
Open SG without BR	458	13	(2.8)	0		23	(5.0)	39	5	(12.8)	0		7	(17.9)	7	111	5	4	46	8
Arch TEVAR with BR	1086	21	(1.9)	4		43	(4.0)	67	7	(10.4)	0		10	(14.9)	0	0	0	0	3	2
Thoracoabdominal TEVAR with BR	127	12	(9.4)	2	(1.6)	14	(11.0)	11	2	(18.2)	0		3	(27.3)	0	0	0	0	0	0
Other	103	3	(2.9)	0		9	(8.7)	21	1	(4.8)	0		2	(9.5)	1	10	2	3	6	4
Total	11,283	264	(2.3)	17	(0.2)	434	(3.8)	855	103	(12.0)	4	(0.5)	153	(17.9)	427	2959	190	98	813	278
(), % mortality																				
Ao, aorta; AVP, aortic valve repair; AVR, aortic valve replacement; MVP, mitral valve repair; MVR, mitral valve replacement; CABG. coronary artery bypass grafting; TEVAR, thoracic endovascular aortic(aneurysm) repair

**Table 4:** Pulmonary thromboembolism (total; 177)

	Cases	30-day mortality				Hospital mortality	
		Hospital		After discharge			
Acute	125	14	(11.2)	0		20	(16.0)
Chronic	52	1	(1.9)	0		1	(1.9)
Total	177	15	(8.5)	0		21	(11.9)

(), % mortality

**Table 5:** Implantation of VAD (total; 156)

	Cases	30-day mortality				Hospital mortality	
		Hospital		After discharge			
Implantation of VAD	156	1	(0.6)	0		10	(6.4)

(), % mortality

VAD, ventricular assist devise

**Table 6:** Heart transplantation (total; 111)

	Cases	Hospital mortality	
Heart transplantation	111	2	(1.8)
Heart and lung transplantation	0	0	
Total	111	2	(1.8)

(), % mortality

Among the 7866 procedures for congenital heart disease conducted in 2024, 6022 were open-heart surgeries, with an overall hospital mortality rate of 1.9% (Table 1). The number of surgeries for neonates and infants in 2024 significantly decreased compared to that in 2014 (3704 vs 4910); on the other hands, hospital mortality did not significantly differ compared to those in 2014 (6.9% vs. 4.9% for neonates and 2.7% vs. 2.4% for infants) despite the increasing ratio of surgeries for severe cases. In 2024, atrial septal defect (1212 cases) and ventricular septal defect (1215 cases) were the most common diseases as previously reported, with patients aged ≥ 18 years accounting for 40% of atrial septal defect and ventricular septal defect surgeries.

Hospital mortality of open heart surgeries for complex congenital heart disease within the past 10 years was as follows (2014 [8], 2019 [2], and 2024): complete atrioventricular septal defect (1.7%, 1.4%, and 2.7%); tetralogy of Fallot (1.1%, 0.7%, and 2.0%); transposition of the great arteries with the intact septum (6.6%, 1.9%, and 6.6%), ventricular septal defect (3.9%, 1.8%, and 4.3%), single ventricle (4.3%, 3.5%, and 3.5%); and hypoplastic left heart syndrome (9.8%, 7.4%, and 4.8%). Currently, right heart bypass surgery has been commonly performed (253bidirectional Glenn procedures, excluding 22 Damus–Kaye–Stansel procedures, and 255 Fontan type procedures, including total cavopulmonary connection) with acceptable hospital mortality rates (0% and 0.8%). The Norwood type I procedure was performed in 63 cases, with a relatively low hospital mortality rate (6.3%) (Table 1).

Valvular heart disease procedures were performed less than that in the previous year. Isolated aortic valve replacement/repair with/without coronary artery bypass grafting (CABG) (n = 7711) was 2.3% less than that in the previous year (n = 7893) and 24.9% fewer than that 5 years ago (n = 10,268 in 2019), as opposed to the rapid increase of transcatheter aortic valve replacement (n = 15,019 and 16,261 in 2023 and 2024). Isolated mitral valve replacement/repairs with/without CABG (n = 5393) was 5.2% more than that in the previous year (n = 5126) and 2.9% more than that 5 years ago (n = 5239 in 2019). Aortic and mitral valve replacement with bioprosthesis were performed in 8182 and 2352 cases, respectively. The rate at which bioprosthesis was used had dramatically increased from 30% in the early 2000s [9, 10] to 87.2% and 77.7% in 2024 for aortic and mitral positions, respectively. Additionally, CABG was performed concurrently in 14.7% of all valvular procedures (17.3% in 2014 [8] and 16.5% in 2019 [2]). Valve repair was common in mitral and tricuspid valve positions (6387 and 3490 cases, respectively) but less common in aortic valve positions (155 patients, only 1.6% of all aortic valve procedures). Mitral valve repair accounted for 67.4% of all mitral valve procedures. Hospital mortality rates for isolated valve replacement for aortic and mitral positions were 2.5% and 6.9%, respectively, but only 1.3% for mitral valve repair. Moreover, hospital mortality rates for redo isolated valve surgery for the aortic and mitral positions were 5.9% and 5.7%, respectively. Finally, overall hospital mortality rates did not significantly improve over the past 10 years (3.1% in 2014 [8], 3.3% in 2019 [2], and 2.6% in 2024) (Table 2).

Isolated CABG had been performed in 9840 cases, accounting for only 68.1% of the procedures performed 10 years ago (n = 14,454 in 2014) [8]. Of the aforementioned cases, 5612 (57.0%) underwent off-pump CABG, with a success rate of 97.7%. The percentage of planned off-pump CABG in 2024 was similar to that in 2023 [7]. Hospital mortality associated with primary elective CABG procedures among 8,565 cases accounted for 2.1%, which is slightly higher than that in 2014 (1.3%) [8]. Hospital mortality for primary emergency CABG among 1,189 cases remained high (10.1%). The percentage of conversion from off-pump to on-pump CABG or on-pump beating-heart CABG was 2.1% among the primary elective CABG cases, with a hospital mortality rate of 14.4%. Patients with end-stage renal failure on dialysis had higher hospital mortality rates than overall mortality, regardless of surgical procedure (on-pump arrest, on-pump beating, and off-pump). This study excluded concomitant CABGs alongside other major procedures under the ischemic heart disease category but rather under other categories, such as valvular heart disease and thoracic aortic aneurysm. Accordingly, the overall number of CABGs in 2023, including concomitant CABG with other major procedures, was 14,209 (Table 2).

Arrhythmia management was primarily or concomitantly performed in 6507 cases, with a hospital mortality rate of 3.2%. Pacemaker and implantable cardioverter-defibrillator implantation were not included in this category (Table 2).

In 2024, 24,333 procedures for thoracic and thoracoabdominal aortic diseases were performed, among which aortic dissection and non-dissection accounted for 12,195 and 12,138, respectively. The number of surgeries for aortic dissection this year was 2.3% higher than that in the preceding year (n = 11,917 in 2023). Hospital mortality rates for the 7158 Stanford type A acute aortic dissections remained high (10.1%). The number of procedures for non-aortic dissections increased by 1.9%, with a hospital mortality rate of 4.8% for all aneurysms and 3.8% and 17.9% for unruptured and ruptured aneurysms, respectively. Thoracic endovascular aortic repair (TEVAR) has been performed for aortic diseases at an increasing rate [2–5, 7]. Stent graft placement was performed in 6131 patients with aortic dissection, including 2865 TEVARs and 3266 open stent graftings. Moreover, 1690 and 372 cases underwent TEVAR and open stent grafting for type B chronic aortic dissection, accounting for 59.0% and 11.4% of the total number of cases, respectively. Hospital mortality rates associated with simple TEVAR for type B aortic dissection were 10.3% and 1.6% for acute and chronic cases, respectively. Stent graft placement was performed in 6179 patients with non-dissected aortic aneurysms, among which 4089 were TEVARs (almost the same number compared to that in 2023, n = 4090) and 2090 were open stent graftings (a 15.3% increase compared to that in 2023, n = 1812). Hospital mortality rates were 3.3% and 17.5% for TEVARs and 4.4% and 21.4% for open stenting in unruptured and ruptured aneurysms, respectively (Table 3).

### (B) General thoracic surgery

**Fig. 2:**
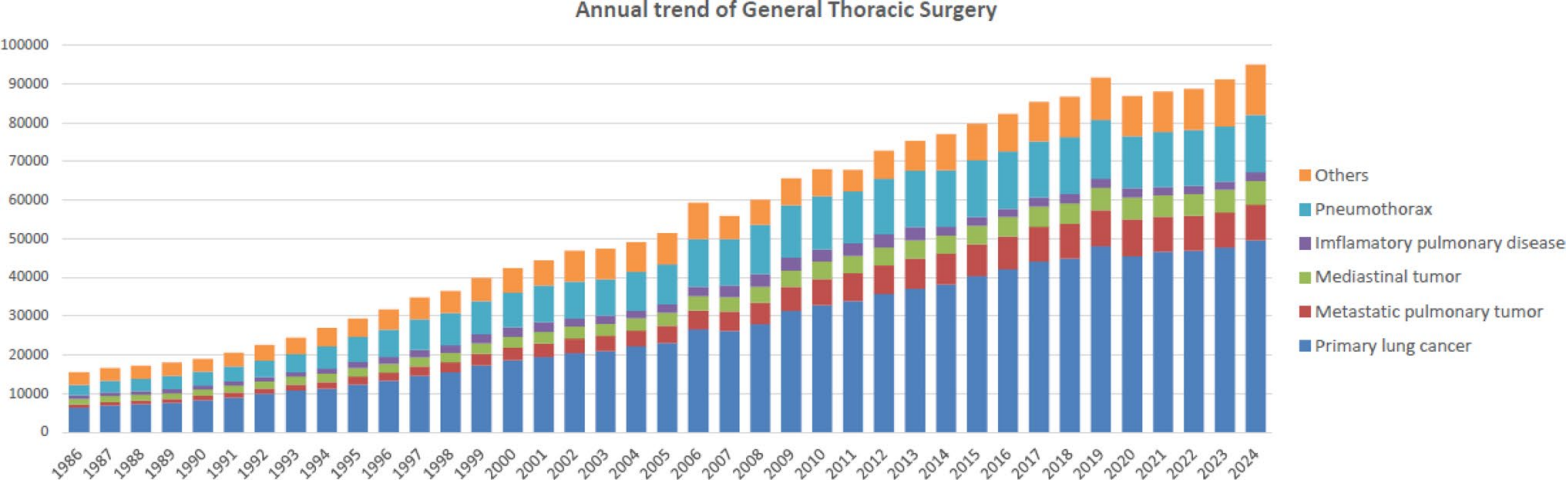
Annual trend of general thoracic surgery

The 2024 survey of general thoracic surgeries comprised 700 surgical units, with bulk data submitted via a web-based collection system established by the NCD [4]. General thoracic surgery departments reported 94,961 procedures in 2024 (Table 7), which is 2.2 times more than that in 2000 and 3335 more procedures than that in 2019 [2] (Fig. 2). It increased compared to that in 2020 (the first year of COVID-19 pandemic: 86,813) [3] by 9.4% and was above the level of 2019 (before COVID-19 pandemic: 91,626) [2].

**Table 7:** Total cases of general thoracic surgery during 2024

	Cases	%
Benign pulmonary tumor	2,606	2.7
Primary lung cancer	49,541	52.2
Other primary malignant pulmonary tumor	465	0.5
Metastatic pulmonary tumor	9,294	9.8
Tracheal tumor	98	0.1
Pleural tumor including mesothelioma	543	0.6
Chest wall tumor	645	0.7
Mediastinal tumor	6,149	6.5
Thymectomy for MG without thymoma	86	0.1
Inflammatory pulmonary disease	2,262	2.4
Empyema	5,143	5.4
Bullous disease excluding pneumothorax	298	0.3
Pneumothorax	14,719	15.5
Chest wall deformity	344	0.4
Diaphragmatic hernia including traumatic	31	0.0
Chest trauma excluding diaphragmatic hernia	637	0.7
Lung transplantation	148	0.2
Others	1,952	2.1
Total	94,961	100.0

In 2024, 49,541 procedures for primary lung cancer had been performed which increased by 3.9% compared to that of 2023 (47,659) [7], and was above the level of 2019 (48,052) [2], similarly to the total number of surgeries in general thoracic surgery. The procedures for lung cancer account for 52% of all general thoracic surgeries in 2024.

Information about the number of video-assisted thoracoscopic surgery (VATS), which is defined as surgical procedures using a skin incision less than 8 cm including a mini-thoracotomy (hybrid) approach, have been available since the 2015 annual report. Tables 8, 9, 11, 14, 15, 16, 18, 19, 20, 21, 22, 24, 25, 26 present the number of VATS procedures for benign pulmonary tumors, primary lung cancer, metastatic pulmonary tumor, chest wall tumor, mediastinal tumor, thymectomy for myasthenia gravis, inflammatory pulmonary disease, empyema, descending necrotizing mediastinitis, bullous diseases, pneumothorax, diaphragmatic hernia, chest trauma and other respiratory surgeries in 2024, respectively.

**Table 8:** Benign pulmonary tumor

	Cases	30-Day mortality				Hospital mortality		by VATS
		Hospital		After discharge				
1. Benign pulmonary tumor								
Hamartoma	498	0		0		0		488
Sclerosing hemangioma	93	0		0		0		89
Papilloma	35	0		0		0		30
Mucous gland adenoma bronchial	35	0		0		0		35
Fibroma	154	0		0		0		148
Lipoma	8	0		0		0		8
Neurogenic tumor	21	0		0		0		20
Clear cell tumor	3	0		0		0		3
Leiomyoma	17	0		0		0		16
Chondroma	6	0		0		0		6
Inflammatory myofibroblastic tumor	0	0		0		0		0
Pseudolymphoma	18	0		0		0		18
Histiocytosis	21	0		0		0		19
Teratoma	9	0		0		0		7
Others	1688	4	(0.2)	0		6	(0.4)	1588
Total	2,606	4	(0.15)	0		6	(0.23)	2,475

(), Mortality %

**Table 9:** Primary malignant pulmonary tumor

	Cases	30-Day mortality				Hospital mortality		VATS	Robotic surgery
		Hospital		After discharge					
2. Primary malignant pulmonary tumor	50,006	101	(0.2)	51	(0.1)	225	(0.4)	34,985	9,172
Lung cancer	49,541	100	(0.2)	51	(0.1)	220	(0.4)	34,985	9,172
Histological classification									
Adenocarcinoma	35,104	42	(0.1)	21	(0.06)	81	(0.2)		
Squamous cell carcinoma	8457	40	(0.5)	17	(0.2)	102	(1.2)		
Large cell carcinoma	312	0		0		0			
LCNEC	522	2	(0.4)	1	(0.2)	3	(0.6)		
Small cell carcinoma	837	4	(0.5)	2	(0.2)	9	(1.1)		
Adenosquamous carcinoma	550	3	(0.5)	1	(0.2)	5	(0.9)		
Carcinoma with pleomorphic, sarcomatoid or sarcomatous elements	542	2	(0.4)	4	(0.7)	2	(0.4)		
Carcinoid	274	0		0		0			
Carcinomas of salivary-gland type	44	0		0		0			
Unclassified	58	0		0		2	(3.4)		
Multiple lung cancer	2225	3	(0.1)	3	(0.1)	8	(0.4)		
Others	570	4	(0.7)	2	(0.4)	8	(1.4)		
Operative procedure									
Wedge resection	9951	17	(0.2)	11	(0.1)	29	(0.3)	9428	36
Segmental excision	11,081	13	(0.1)	5	(0.05)	21	(0.2)	7909	2586
(Sleeve segmental excision)	15	0		0		0		9	1
Lobectomy	28,080	59	(0.2)	31	(0.11)	154	(0.5)	17,434	6540
(Sleeve lobectomy)	327	0		2	(0.6)	2	(0.6)	52	37
Pneumonectomy	139	5	(3.6)	1	(0.7)	7	(5.0)	11	6
(Sleeve pneumonectomy)	3	0		0		0		0	0
Other bronchoplasty	27	0		1	(3.7)	1	(3.7)	2	1
Pleuropneumonectomy	0	0		0		0		0	0
Others	217	6	(2.8)	2	(0.9)	8	(3.7)	157	3
Multiple incision for multiple lung cancer	46	0		1	(2.2)	0		44	0
Sarcoma	44	1	(2.3)	0		2	(4.5)		
AAH	122	0		0		0			
Lymphoma	216	0		0		2	(0.9)		
Others	83	0		0		1	(1.2)		

(), Mortality %

A total of 2606 procedures for benign pulmonary tumors had been conducted in 2024 (Table 8). Hamartomas were the most frequent benign pulmonary tumors diagnosed, with 2475 patients (95%) undergoing VATS.

Tables 9 and 10 show additional information on primary malignant pulmonary tumors. Accordingly, the most frequently diagnosed lung cancer subtype was adenocarcinoma (71% of all lung cancers), followed by squamous cell carcinoma (17%). Sublobar resection was performed in 21,032 lung cancer cases (42% of all cases) and lobectomy in 28,080 cases (57% of all cases). Sleeve lobectomy was performed in 327 cases (0.7% of all cases), while pneumonectomy was required in 139 cases (0.3% of all cases). VATS lobectomy was performed in 17,434 cases of lung cancer (62% of all lobectomy cases). RATS lobectomy was performed in 6540 cases of lung cancer (23% of all lobectomy cases). Patients aged ≥ 80 years who underwent lung cancer surgery accounted for 9012 (18%). Among those who died within 30 days postoperatively, 100 and 51 died before and after hospital discharge, respectively. Overall, 151 patients died within 30 days postoperatively (30-day mortality rate, 0.3%), while 220 died before discharge (hospital mortality rate, 0.4%). Moreover, 30-day mortality rates according to the procedure were 0.2%, 0.3%, and 4.3% for segmentectomy, lobectomy, and pneumonectomy, respectively. Interstitial pneumonia had been the leading cause of death after lung cancer surgery, followed by pneumonia, cardiovascular events, respiratory failure, bronchopleural fistule, and brain infarction or bleeding.

**Table 10:** Details of lung cancer operations

TNM	
c-Stage	Cases
0	2311
IA1	9698
IA2	14,821
IA3	8746
IB	5043
IIA	1709
IIB	3662
IIIA	2402
IIIB	414
IIIC	21
IVA	444
IVB	106
NA	117
Total	49,494

p-Stage	Cases
0(pCR)	3291
IA1	10,029
IA2	11,914
IA3	5804
IB	6996
IIA	1401
IIB	4360
IIIA	3469
IIIB	616
IIIC	13
IVA	950
IVB	106
NA	546
Total	49,495

Sex	Cases
Male	29,217
Female	20,278
NA	0
Total	49,495

Age (y)	Cases
< 20	23
20–29	59
30–39	246
40–49	1158
50–59	3881
60–69	10,643
70–79	24,470
80–89	8864
≥ 90	148
NA	3
Total	49,495

Cause of death	Cases
Cardiovascular	46
Pneumonia	68
Pyothorax	3
Bronchopleural fistula	10
Respiratory failure	32
Pulmonary embolism	2
Interstitial pneumonia	98
Brain infarction or bleeding	10
Others	143
Unknown	36
Total	448

The procedures for metastatic pulmonary tumors performed in 2024 (9294) increased 1.7% compared to that in 2023 (9140) [5] (Table 11). Among such procedures, the most frequent primary tumor was colorectal cancer (48% of all cases).

**Table 11:** Metastatic pulmonary tumor

	Cases	30-Day mortality				Hospital mortality		VATS	Robotic surgery
		Hospital		After discharge					
3. Metastatic pulmonary tumor	9,294	10	(0.1)	10	(0.11)	19	(0.20)	8,689	704
Colorectal	4437	3	(0.07)	4	(0.09)	7	(0.16)	4154	366
Hepatobiliary/Pancreatic	552	1	(0.2)	1	(0.2)	1	(0.18)	533	38
Uterine	528	0		1	(0.2)	0		496	57
Mammary	569	0		0		1	(0.18)	551	37
Ovarian	81	0		0		0		77	9
Testicular	31	0		0		0		27	2
Renal	742	1	(0.1)	1	(0.1)	2	(0.27)	699	56
Skeletal	104	0		0		0		97	5
Soft tissue	255	0		0		0		218	15
Otorhinolaryngological	498	1	(0.2)	0		1	(0.20)	470	27
Pulmonary	427	1	(0.2)	1	(0.2)	4	(0.94)	370	17
Others	1070	3	(0.3)	2	(0.2)	3	(0.28)	997	75

(), Mortality %

A total of 98 procedures for tracheal tumors, including 40, 36, and 22 cases of primary malignant, metastatic, and benign tracheal tumors, respectively, were performed in 2024. Further, 17 patients underwent sleeve resection and reconstruction (Table 12).

**Table 12:** Tracheal tumor

	Cases	30-Day mortality				Hospital mortality	
		Hospital		After discharge			
4. Tracheal tumor	98	4	(4.1)	0		7	(7.1)
A. Primary malignant tumor							
Histological classification							
Squamous cell carcinoma	7	0		0		0	
Adenoid cystic carcinoma	15	0		0		0	
Mucoepidermoid carcinoma	3	0		0		0	
Others	15	1	(6.7)	0		1	(6.7)
Total	40	1	(2.5)	0		1	(2.5)
B. Metastatic/invasive malignant tumor e.g. invasion of thyroid cancer							
	36	3	(8.3)	0		6	(16.7)
C. Benign tracheal tumor							
Histological classification							
Papilloma	0	0		0		0	
Adenoma	2	0		0		0	
Neurofibroma	1	0		0		0	
Chondroma	0	0		0		0	
Leiomyoma	1	0		0		0	
Others	18	0		0		0	
Histology unknown	0	0		0		0	
Total	22	0		0		0	
Operation							
Sleeve resection with reconstruction	17	0		0		1	(5.9)
Wedge with simple closure	0	0		0		0	
Wedge with patch closure	0	0		0		0	
Total laryngectomy with tracheostomy	0	0		0		0	
Others	5	1	(20.0)	0		1	(20.0)
Unknown	0	0		0		0	
Total	22	1	(4.5)	0		2	(9.1)

(), Mortality %

Overall, 543 pleural tumors had been diagnosed in 2024 (Table 13), with diffuse malignant pleural mesothelioma as the most frequent histologic diagnosis. Total pleurectomy was performed in 99 cases and extrapleural pneumonectomy in 15 cases. The 30-day mortality rate were 1% and 6.7% after total pleurectomy and extrapleural pneumonectomy, respectively.

**Table 13:** Tumor of pleural origin

5. Tumor of pleural origin							
Histological classification	Cases	30-Day mortality				Hospital mortality	
		Hospital		After discharge			
Solitary fibrous tumor	124	0		0		1	(0.8)
Diffuse malignant pleural mesothelioma	168	2	(1.2)	2	(1.2)	7	(4.2)
Localized malignant pleural mesothelioma	28	0		0		0	
Others	223	3	(1.3)	0		4	(1.8)
Total	543	5	(0.9)	2	(0.4)	12	(2.2)

Operative procedure	Cases	30-Day mortality				Hospital mortality	
		Hospital		After discharge			
Extrapleural pneumonectomy	15	0		1	(6.7)	0	
Total pleurectomy	99	1	(1.0)	0		4	(4.0)
Others	54	1	(1.9)	1	(1.9)	3	(5.6)
Total	168	2	(1.2)	2	(1.2)	7	(4.2)

(), Mortality %

Overall, 645 chest wall tumor resections had been performed in 2024, including 105, 138, and 402 cases of primary malignant, metastatic, and benign tumors, respectively (Table 14).

**Table 14:** Chest wall tumor

6. Chest wall tumor								
	Cases	30-Day mortality				Hospital mortality		VATS
		Hospital		After discharge				
Primary malignant tumor	105	0		0		0		34
Metastatic malignant tumor	138	0		0		2	(1.4)	45
Benign tumor	402	0		0		0		317
Total	645	0		0		2	(0.3)	396

(), Mortality %

In 2024, 6149 mediastinal tumors were resected, which increased by 5.0% that in 2023 (5851) (Table 15) [7]. Thymic epithelial tumors, including 2,423 thymomas, 400 thymic carcinomas, and 58 thymic carcinoids, were the most frequently diagnosed mediastinal tumor subtypes.

**Table 15:** Mediastinal tumor

	Cases	30-Day mortality				Hospital mortality		By VATS	Robotic surgery
		Hospital		After discharge					
7. Mediastinal tumor	6,149	5	(0.08)	3	(0.05)	10	(0.2)	5,033	2,320
Thymoma	2423	3	(0.1)	0		4	(0.2)	1878	963
Thymic cancer	400	1	(0.3)	1	(0.3)	1	(0.3)	268	140
Thymus carcinoid	58	0		0		0		33	23
Germ cell tumor	81	0		0		0		51	22
Benign	65	0		0		0		47	21
Malignant	16	0		0		0		4	1
Neurogenic tumor	499	0		0		1	(0.2)	463	180
Congenital cyst	1339	0		0		0		1287	601
Goiter	83	0		0		2	(2.4)	35	11
Lymphatic tumor	223	0		1	(0.4)	1	(0.4)	185	76
Excision of pleural recurrence of thymoma	63	0		0		0		41	6
Thymolipoma	21	0		0		0		18	3
Others	959	1	(0.1)	1	(0.1)	1	(0.1)	774	295

(), Mortality %

A total of 486 patients underwent thymectomy for myasthenia gravis (Table 16), among which 400 procedures (82%) were associated with thymoma in 2024.

**Table 16:** Thymectomy for myasthenia gravis

	Cases	30-Day mortality				Hospital mortality		by VATS	Robotic surgery
		Hospital		After discharge					
8. Thymectomy for myasthenia gravis	486	1	(0.2)	0		1	(0.2)	355	201
With thymoma	400	1	(0.3)	0		1	(0.3)	287	170

(), Mortality %

Overall, 25,385 patients underwent procedures for non-neoplastic disease in 2024. Accordingly, 2262 patients underwent lung resection for inflammatory lung diseases (Tables 17 and 18), among which 414 and 267 patients were associated with mycobacterial and fungal infections, respectively. Procedures for inflammatory pseudotumor were performed in 1,047 cases (46%).

**Table 17:** Operations for non-neoplastic diseases:A + B + C + D + E + F + G + H + I

	Cases	30-Day mortality				Hospital mortality	
		Hospital		After discharge			
9. Operations for non-neoplastic diseases	25,385	306	(1.2)	48	(0.2)	645	(2.5)

**Table 18:** A. Inflammatory pulmonary disease

	Cases	30-Day mortality				Hospital mortality		VATS
		Hospital		After discharge				
A. Inflammatory pulmonary disease	2,262	11	(0.5)	1	(0.0)	19	(0.8)	2,002
Tuberculous infection	27	0		0		0		24
Mycobacterial infection	414	1	(0.2)	0		1	(0.2)	363
Fungal infection	267	8	(3.0)	0		13	(4.9)	183
Bronchiectasis	42	1	(2.4)	0		1	(2.4)	32
Tuberculous nodule	38	0		0		0		36
Inflammatory pseudotumor	1047	0		0		2	(0.2)	994
Interpulmonary lymph node	54	0		0		0		54
Others	373	1	(0.3)	1	(0.3)	2	(0.5)	316

(), Mortality %

A total of 5143 procedures were performed for empyema (Table 19), among which 4361 (85%) were acute and 782 (15%) were chronic. Further, pleural fistulas developed in 568 and 313 patients with acute and chronic empyema, respectively. The hospital mortality rate was 16.9% among patients with acute empyema with fistula.

**Table 19:** B. Empyema

	Cases	30-Day mortality				Hospital mortality		by VATS
		Hospital		After discharge				
Acute empyema	4361	83	(1.9)	13	(0.3)	205	(4.7)	3839
With fistula	568	28	(4.9)	1	(0.2)	96	(16.9)	327
Without fistula	3751	55	(1.5)	12	(0.3)	109	(2.9)	3475
Unknown	42	0		0		0		37
Chronic empyema	782	25	(3.2)	5	(0.6)	69	(8.8)	488
With fistula	313	14	(4.5)	1	(0.3)	37	(11.8)	142
Without fistula	407	8	(2.0)	2	(0.5)	28	(6.9)	298
Unknown	62	3	(4.8)	2	(3.2)	4	(6.5)	48
Total	5,143	108	(2.1)	18	(0.3)	274	(5.3)	4,327

(), Mortality %

Further, 125 operations were performed for descending necrotizing mediastinitis (Table 20), with a hospital mortality rate of 12%.

**Table 20:** C. Descending necrotizing mediastinitis

	Cases	30-Day mortality				Hospital mortality		VATS
		Hospital		After discharge				
C. Descending necrotizing mediastinitis	125	7	(5.6)	0		15	(12.0)	92

(), Mortality %

A total of 298 procedures were conducted for bullous diseases (Table 21), while 18 patients underwent lung volume reduction surgery.

**Table 21:** D. Bullous diseases

	Cases	30-Day mortality				Hospital mortality		VATS
		Hospital		After discharge				
D. Bullous diseases	298	2	(0.7)	0		2	(0.7)	272
Emphysematous bulla	208	2	(1.0)	0		2	(1.0)	189
Bronchogenic cyst	15	0		0		0		13
Emphysema with LVRS	18	0		0		0		16
Others	57	0		0		0		54

(), Mortality %

LVRS, lung volume reduction surgery

A total of 14,719 procedures were performed for pneumothorax (Table 22). Among the 9920 procedures for spontaneous pneumothorax, 2293 (23%) were bullectomies alone, while 6950 (70%) required additional procedures, such as coverage with artificial material, as well as parietal pleurectomy. A total of 4799 procedures for secondary pneumothorax were performed, with chronic obstructive pulmonary disease (COPD) being the most prevalent associated disease (3260 cases, 68%). The hospital mortality rate for secondary pneumothorax associated with COPD was 2.4%.

**Table 22:** E. Pneumothorax

Cases	30-Day mortality				Hospital mortality		VATS
	Hospital		After discharge				
14,719	111	(0.8)	19	(0.1)	200	(1.4)	14,315

Spontaneous pneumothorax								
Operative procedure	Cases	30-Day mortality				Hospital mortality		VATS
		Hospital		After discharge				
Bullectomy	2,293	0		0		2	(0.1)	2,253
Bullectomy with additional procedure	6,950	10	(0.1)	2	(0.03)	17	(0.2)	6,857
Coverage with artificial material	6789	9	(0.1)	2	(0.03)	16	(0.2)	6,699
Parietal pleurectomy	13	0		0		0		12
Coverage and parietal pleurectomy	48	0		0		0		47
Others	100	1	(1.0)	0		1	(1.0)	99
Others	675	5	(0.7)	0		8	(1.2)	625
Unknown	2	0		0		0		1
Total	9,920	15	(0.2)	2	(0.0)	27	(0.3)	9,736

Secondary pneumothorax								
Associated disease	Cases	30-Day mortality				Hospital mortality		VATS
		Hospital		After discharge				
COPD	3260	44	(1.3)	7	(0.2)	77	(2.4)	3148
Tumorous disease	174	8	(4.6)	4	(2.3)	14	(8.0)	166
Catamenial	222	1	(0.5)	0		2	(0.9)	218
LAM	28	0		0		0		27
Others (excluding pneumothorax by trauma)	1115	43	(3.9)	6	(0.5)	80	(7.2)	1020
Unknown	0	0		0		0		0

Operative procedure	Cases	30 Day mortality				Hospital mortality		VATS
		Hospital		After discharge				
Bullectomy	906	12	(1.3)	6	(0.7)	25	(2.8)	886
Bullectomy with additional procedure	2,841	45	(1.6)	8	(0.3)	69	(2.4)	2,757
Coverage with artificial material	2734	44	(1.6)	8	(0.3)	67	(2.5)	2656
Parietal pleurectomy	8	0		0		0		8
Coverage and parietal pleurectomy	24	0		0		1	(4.2)	23
Others	75	1	(1.3)	0		1	(1.3)	70
Others	1,050	39	(3.7)	3	(0.3)	79	(7.5)	935
Unknown	2	0		0		0		1
Total	4,799	96	(2.0)	17	(0.4)	173	(3.6)	4,579

(), Mortality %.

The 2024 survey reported 344 procedures for chest wall deformity (Table 23). However, this may have been underestimated because the Nuss procedure for pectus excavatum was more likely performed in pediatric surgery centers not associated with the Japanese Association for Thoracic Surgery.

**Table 23:** F. Chest wall deformity

	Cases	30-Day mortality				Hospital mortality	
		Hospital		After discharge			
F. Chest wall deformity	344	1	(0.3)	0		0	
Funnel chest	334	0		0		0	
Others	10	1	(10.0)	0		0	

(), Mortality %

Surgical treatment for diaphragmatic hernia was performed in 31 patients (Table 24). This may have been underestimated because procedures may have been classified as gastrointestinal surgery.

**Table 24:** G. Diaphragmatic hernia

	Cases	30-Day mortality				Hospital mortality		VATS
		Hospital		After discharge				
G. Diaphragmatic hernia	31	0		0		0		9
Congenital	6	0		0		0		0
Traumatic	10	0		0		0		4
Others	15	0		0		0		5

(), Mortality %

The survey reported 637 procedures for chest trauma, excluding iatrogenic injuries (Table 25), with a hospital mortality rate of 6.3%.

**Table 25:** H. Chest trauma

	Cases	30-Day mortality				Hospital mortality		VATS
		Hospital		After discharge				
H. Chest trauma	637	27	(4.2)	2	(0.3)	40	(6.3)	392

(), Mortality %

Table 26 summarizes the procedures for other diseases, including 124 and 129 cases of arteriovenous malformation and pulmonary sequestration, respectively.

**Table 26:** I. Other respiratory surgery

	Cases	30-Day mortality				Hospital mortality		VATS
		Hospital		After discharge				
I. Other respiratory surgery	1827	40	(2.2)	8	(0.4)	94	(5.1)	1362
Arteriovenous malformation	124	1	(0.8)	0		1	(0.8)	120
Pulmonary sequestration	129	0		0		0		115
Postoperative bleeding ・air leakage	586	19	(3.2)	5	(0.9)	49	(8.4)	406
Chylothorax	53	1	(1.9)	0		3	(5.7)	46
Others	935	19	(2.0)	3	(0.3)	41	(4.4)	675

(), Mortality %

A total of 148 lung transplantations were performed in 2024 which increased by 17% compared to 127 in 2023 [7] (Table 27), among which 107 and 34 were from brain-dead and living-related donors, respectively. 30-day mortality for total lung transplantation was 5.4% (8/148).

**Table 27:** Lung transplantation

	Cases	30-Day mortality				Hospital mortality	
		Hospital		After discharge			
Single lung transplantation from brain-dead donor	66	2	(3.0)	0		3	(4.5)
Bilateral lung transplantation from brain-dead donor	64	2	(3.1)	0		5	(7.8)
Lung transplantation from living donor	18	0		0		0	
Total lung transplantation	148	4	(2.7)	0		8	(5.4)
Donor of living donor lung transplantation	34	0		0		0	
Donor of brain-dead donor lung transplantation	107						

(), Mortality %

In 2024, the number of VATS procedures increased by 5.9% from 80,320 (2023) to 85,067. The population of VATS procedures in all procedures 90% in 2024 increased by 3% compared to that in 2023 (87%) (Table 28).

**Table 28:** Video-assisted thoracic surgery

	Cases	30-Day mortality				Hospital mortality	
		Hospital		After discharge			
11. Video-assisted thoracic surgery	85,067	299	(0.4)	93	(0.1)	603	(0.7)

(), Mortality % (including thoracic sympathectomy 236)

A total of 610 tracheobronchoplasty procedures were performed in 2024, including 333 sleeve lobectomies, 16 carinal reconstructions and 6 sleeve pneumonectomies (Table 29). Hospital mortality rates for sleeve lobectomy, carinal reconstruction and sleeve pneumonectomy were 0.9, 0 and 0% respectively.

**Table 29:** Tracheobronchoplasty

	Cases	30-Day mortality				Hospital mortality	
		Hospital		After discharge			
12. Tracheobronchoplasty	610	7	(1.1)	4	(0.7)	18	(3.0)
Trachea	32	1	(3.1)	0		2	(6.3)
Sleeve resection with reconstruction	18	0		0		1	(5.6)
Wedge with simple closure	0	0		0		0	
Wedge with patch closure	0	0		0		0	
Total laryngectomy with tracheostomy	0	0		0		0	
Others	14	1	(7.1)	0		1	(7.1)
Carinal reconstruction	16	0		0		0	
Sleeve pneumonectomy	6	0		0		0	
Sleeve lobectomy	333	0		2	(0.6)	3	(0.9)
Sleeve segmental excision	19	0		0		0	
Bronchoplasty without lung resection	15	0		0		0	
Others	189	6	(3.2)	2	(1.1)	13	(6.9)

(), Mortality %

A total of 368 pediatric surgery was performed in 2024 with hospital mortality of 2.2% (Table 30).

**Table 30:** Pediatric surgery

	Cases	30-Day mortality				Hospital mortality	
		Hospital		After discharge			
13. Pediatric surgery	368	8	(2.2)	1	(0.3)	8	(2.2)

(), Mortality %

Overall, 1,169 combined resection of neighboring organ(s) had been performed for primary lung cancer and mediastinal tumor in 2024. The combined resection for primary lung cancer includes 219, 90, 73, 52, 20, 12, 11, and 5 cases of chest wall, pulmonary artery, pericardium, diaphragm, superior vena cava, brachiocephalic vein, left atrium, and esophagus resections, respectively. The combines resection for mediastinal tumor includes 494, 323, 136, 52, 43 and 12 cases of lung, pericardium, brachiocephalic vein, superior vena cava, diaphragm and chest wall resections, respectively (Table 31).

**Table 31:** Combined resection of neighboring organ(s)

	Cases	30-Day mortality				Hospital mortality	
		Hospital		After discharge			
14. Combined resection of neighboring organ(s)	1169	5	(0.4)	2	(0.2)	11	(0.9)

Organ resected	Cases	30-Day mortality				Hospital mortality	
		Hospital		After discharge			
A. Primary lung cancer							
Aorta	2	0		0		0	
Superior vena cava	20	1	(5.0)	0		2	(10.0)
Brachiocephalic vein	12	0		0		0	
Pericardium	73	0		1	(1.4)	0	
Pulmonary artery	90	0		0		2	(2.2)
Left atrium	11	0		0		0	
Diaphragm	52	1	(1.9)	0		3	(5.8)
Chest wall (including ribs)	219	2	(0.9)	1	(0.5)	4	(1.8)
Vertebra	3	0		0		0	
Esophagus	5	0		0		0	
Total	487	4	(0.8)	2	(0.4)	11	(2.3)
B. Mediastinal tumor							
Aorta	4	0		0		0	
Superior vena cava	52	0		0		0	
Brachiocephalic vein	136	0		0		0	
Pericardium	323	0		0		0	
Pulmonary artery	3	0		0		0	
Left atrium	1	0		0		0	
Diaphragm	43	0		0		0	
Chest wall (including ribs)	12	1	(8.3)	0		1	(8.3)
Vertebra	6	0		0		0	
Esophagus	6	0		0		0	
Lung	494	1	(0.2)	0		1	(0.2)
Total	1,080	2	(0.2)	0		2	(0.2)

(), Mortality %

A total of 552 operations of lung cancer invading the chest wall of apex had been performed in 2024 with hospital mortality of 1.1% (Table 32).

**Table 32:** Operation of lung cancer invading the chest wall of the apex

	Cases	30-Day mortality				Hospital mortality	
		Hospital		After discharge			
15. Operation of lung cancer invading the chest wall of the apex	552	1	(0.2)	1	(0.2)	6	(1.1)

(), Mortality %

Includes tumors invading the anterior apical chest wall and posterior apical chest wall (superior sulcus tumor, so called Pancoast type)

A total of 5006 diagnostic procedures were performed in 2024 (Table 33).

**Table 33:** Diagnostic procedures

	Cases	30-Day mortality				Hospital mortality	
		Hospital		After discharge			
Mediastinoscopic biopsy	181	0		0		2	(1.1)
Lung biopsy for diffuse parenchymal lung disease	481	1	(0.2)	0		2	(0.4)
Biopsy for lymph node, tumor and pleura	2865	32	(1.1)	13	(0.5)	62	(2.2)
Others	1479	49	(3.3)	15	(1.0)	93	(6.3)

(), Mortality %

### (C) Esophageal surgery

In 2018, the data collection method for esophageal surgery had been modified from self-reports using questionnaire sheets following each institution belonging to the Japanese Association for Thoracic Surgery to an automatic package downloaded from the NCD in Japan. Consequently, the registry excluded data for non-surgical cases with esophageal diseases. Furthermore, data regarding the histological classification of malignant tumors, multiple primary cancers, and mortality rates for cases with combined resection of other organs could not be registered because they were not included in the NCD. Instead, detailed data regarding postoperative surgical and non-surgical complications were collected from the NCD. Moreover, data regarding surgeries for corrosive esophageal strictures and salvage surgeries for esophageal cancer had been exceptionally registered by participating institutions (Table 34).

**Table 34:** Benign esophageal diseases

	Operation( +)							T/L*3						
	Cases	Hospital mortality						Cases	Hospital mortality					
		~ 30 days		31–90 days		Total (including after 91 days mortality)			~ 30 days		31–90 days		Total (including after 91 days mortality)	
1.Achalasia	118	1	(0.8)	0		1	(0.8)	41	1	(2.4)	0		1	(2.4)
2.Benign tumor	41	0		0		0		38	0		0		0	
3.Diverticulum	26	0		0		0		12	0		0		0	
4.Hiatal hernia	535	6	(1.1)	1	(0.2)	7	(1.3)	467	3	(0.6)	1	(0.2)	4	(0.9)
5.Spontaneous rupture of the esophagus	86	5	(5.8)	1	(1.2)	6	(7.0)	2	0		0		0	
6.Esophago-tracheal fistula	2	0		0		0		0	0		0		0	
7.Esophagitis, Esophageal ulcer	73	0		0		0		68	0		0		0	
8.Corrosive stricture of the esophagus	17	0		0		0		16	0		0		0	
Total	898	12	(1.3)	2	(0.2)	14	(1.6)	644	4	(0.6)	1	(0.2)	5	(0.8)

(), Mortality %

T/L: Thoracoscopic and/or laparoscopic

**Fig. 3:**
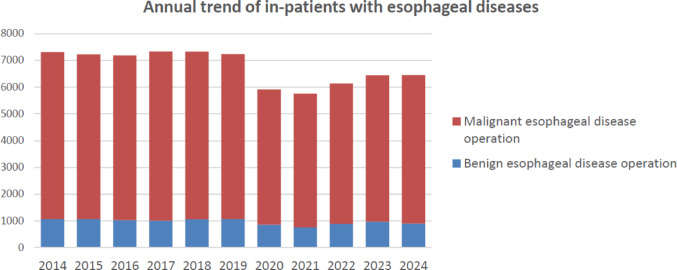
Annual trend of in-patients with esophageal diseases

Throughout 2024, 6,455 patients underwent surgery for esophageal diseases (898 and 5557 for benign and malignant esophageal diseases, respectively) from institutions across Japan (Fig. 3). Compared to 2019, there was a total decrease of 780 cases (10.8%) observed. These significant declines which were largely influenced by the COVID-19 pandemic that began in 2020, with factors such as surgical restrictions, reduced medical visits, and postponed screenings being considered as contributing factors. However, the number of esophageal surgeries in 2024 increased by 16 compared to 2023. As the issues related to COVID-19 are being resolved, a gradual recovery in the number of surgeries is expected in the future.

Concerning benign esophageal diseases (Table 34), thoracoscopic and/or laparoscopic surgeries were performed in 93.2% (68/73), 87.3% (467/535), 92.7% (38/41), and 34.7% (41/118) of patients with esophagitis (including esophageal ulcer), hiatal hernia, benign tumors, and achalasia, respectively. The decrease in the proportion of thoracoscopic and/or laparoscopic surgeries for achalasia is likely due to the gradual adoption of peroral endoscopic myotomy (POEM) in Japan. Conversely, 97.7% (84/86) of patients with spontaneous rupture of the esophagus underwent open surgery. Hospital mortality rates within 30 postoperative days were 1.1% (6/535), 5.8% (5/86) for hiatal hernia and spontaneous rupture of the esophagus, respectively.

The most common tumor location for malignant esophageal diseases was the thoracic esophagus (Table 35). Among the cases with esophageal malignancies, esophagectomy for superficial and advanced cancers was performed in 2131 (38.3%) and 3426 (61.7%), respectively. Hospital mortality rates within 30 days after esophagectomy were 0.4% and 0.9% for patients with superficial and advanced cancer, respectively.

**Table 35:** Malignant Esophageal disease

	Operation (+							Thoracoscopic and/or laparscopic procedure								
	Cases	Hospital mortality						Cases	Conversion to thoracotomy		Hospital mortality					
		~ 30 days		31 ~ 90 days		Total (including after 91 days mortality)					~ 30 days		31–90 days		Total (including after 91 days mortality)	
Location																
(1) Cervical esophagus	144	1	(0.7)	1	(0.7)	2	(1.4)	70	0		1	(1.4)	1	(1.4)	2	(2.9)
(2) Thoracic esophagus	4592	30	(0.7)	17	(0.4)	47	(1.0)	4483	10	(0.2)	27	(0.6)	16	(0.4)	43	(1.0)
(3) Abdominal esophagus	565	3	(0.5)	0		3	(0.5)	522	2	(0.4)	3	(0.6)	0		3	(0.6)
Total	5301	34	(0.6)	18	(0.3)	52	(1.0)	5075	12	(0.2)	31	(0.6)	17	(0.3)	48	(0.9)
Tumor depth																
(A) Superficial cancer(T1)																
(1) Transhiatal esophagectomy	3	0		0		0		0	0		0		0		0	
(2) Mediastinoscopic esophagectomy and reconstruction	88	0		0		0		87	0		0		0		0	
(3) Transthoracic (rt.) esophagectomy and reconstruction	945	5	(0.5)	0		5	(0.5)	910	2	(0.2)	4	(0.4)	0		4	(0.4)
(4) Transthoracic (lt.) esophagectomy and reconstruction	12	0		0		0		7	0		0		0		0	
(5) Cervical esophageal resection and reconstruction	8	0		0		0		0	0		0		0		0	
(6) Robot-assisted esophagectomy and reconstruction	906	3	(0.3)	3	(0.3)	6	(0.7)	888	1	(0.1)	3	(0.3)	3	(0.3)	6	(0.7)
(7) Others	14	0		0		0		0	0		0		0		0	
(8) Esophagectomy without reconstruction	155	1	(0.6)	0		1	(0.6)	75	13	(17.3)	1	(1.3)	0		1	(1.3)
Subtotal	2131	9	(0.4)	3	(0.1)	12	(0.6)	1967	16	(0.8)	8	(0.4)	3	(0.2)	11	(0.6)
(B) Advanced cancer(T2-T4)																
(1) Transhiatal esophagectomy	6	0		0		0		0	0		0		0		0	
(2) Mediastinoscopic esophagectomy and reconstruction	131	1	(0.8)	0		1	(0.8)	131	0		1	(0.8)	0		1	(0.8)
(3) Transthoracic (rt.) esophagectomy and reconstruction	1654	15	(0.9)	12	(0.7)	27	(1.6)	1585	8	(0.5)	14	(0.9)	11	(0.7)	25	(1.6)
(4) Transthoracic (lt.) esophagectomy and reconstruction	29	1	(3.4)	1	(3.4)	2	(6.9)	13	0		0		1	(7.7)	1	(7.7)
(5) Cervical esophageal resection and reconstruction	65	0		0		0		0	0		0		0		0	
(6) Robot-assisted esophagectomy and reconstruction	1415	7	(0.5)	2	(0.1)	9	(0.6)	1398	0		7	(0.5)	2	(0.1)	9	(0.6)
(7) Others	24	1	(4.2)	0		1	(4.2)	8	0		1	(12.5)	0		1	(12.5)
(8) Esophagectomy without reconstruction	102	5	(4.9)	3	(2.9)	8	(7.8)	84	27	(32.1)	3	(3.6)	2	(2.4)	5	(6.0)
Subtotal	3426	30	(0.9)	18	(0.5)	48	(1.4)	3219	35	(1.1)	26	(0.8)	16	(0.5)	42	(1.3)
Total	5557	39	(0.7)	21	(0.4)	60	(1.1)	5186	51	(1.0)	34	(0.7)	19	(0.4)	53	(1.0)

	Cases	Overall morbidity		Morbidity ≥ CD III		Surgical complications											
						Surgical site infection						Anastomotic leakage		Recurrent nerve palsy		Wound dehiscence	
						Superficial incision		Deep incision		Organ space							
Location																	
(1) Cervical esophagus	144	72	(50.0)	31	(21.5)	14	(9.7)	8	(5.6)	12	(8.3)	19	(13.2)	20	(13.9)	3	(2.1)
(2) Thoracic esophagus	4592	2483	(54.1)	984	(21.4)	258	(5.6)	141	(3.1)	263	(5.7)	477	(10.4)	603	(13.1)	46	(1.0)
(3) Abdominal esophagus	565	255	(45.1)	100	(17.7)	27	(4.8)	9	(1.6)	33	(5.8)	66	(11.7)	32	(5.7)	5	(0.9)
Total	5301	2810	(53.0)	1115	(21.0)	299	(5.6)	158	(3.0)	308	(5.8)	562	(10.6)	655	(12.4)	54	(1.0)
Tumor depth																	
(A) Superficial cancer(T1)																	
(1) Transhiatal esophagectomy	3	2	(66.7)	0		1	(33.3)	0		0		0		0		0	
(2) Mediastinoscopic esophagectomy and reconstruction	88	58	(65.9)	23	(26.1)	6	(6.8)	6	(6.8)	9	(10.2)	14	(15.9)	19	(21.6)	3	(3.4)
(3) Transthoracic (rt.) esophagectomy and reconstruction	945	504	(53.3)	187	(19.8)	48	(5.1)	25	(2.6)	51	(5.4)	105	(11.1)	125	(13.2)	7	(0.7)
(4) Transthoracic (lt.) esophagectomy and reconstruction	12	4	(33.3)	2	(16.7)	1	(8.3)	1	(8.3)	2	(16.7)	2	(16.7)	0		0	
(5) Cervical esophageal resection and reconstruction	8	4	(50.0)	2	(25.0)	1	(12.5)	2	(25.0)	1	(12.5)	1	(12.5)	2	(25.0)	1	(12.5)
(6) Robot-assisted esophagectomy and reconstruction	906	461	(50.9)	192	(21.2)	47	(5.2)	33	(3.6)	50	(5.5)	90	(9.9)	113	(12.5)	9	(1.0)
(7) Others	14	4	(28.6)	3	(21.4)	0		0		2	(14.3)	2	(14.3)	1	(7.1)	0	
(8) Esophagectomy without reconstruction	155																
Subtotal	2131	1037	(48.7)	409	(19.2)	104	(4.9)	67	(3.1)	115	(5.4)	214	(10.0)	260	(12.2)	20	(0.9)
(B)Advanced cancer(T2-T4)																	
(1) Transhiatal esophagectomy	6	3	(50.0)	1	(16.7)	0		1	(16.7)	1	(16.7)	1	(16.7)	0		0	
(2) Mediastinoscopic esophagectomy and reconstruction	131	84	(64.1)	35	(26.7)	13	(9.9)	2	(1.5)	7	(5.3)	19	(14.5)	28	(21.4)	2	(1.5)
(3) Transthoracic (rt.) esophagectomy and reconstruction	1654	893	(54.0)	347	(21.0)	90	(5.4)	52	(3.1)	96	(5.8)	185	(11.2)	184	(11.1)	18	(1.1)
(4) Transthoracic (lt.) esophagectomy and reconstruction	29	13	(44.8)	5	(17.2)	2	(6.9)	0		4	(13.8)	4	(13.8)	1	(3.4)	0	
(5) Cervical esophageal resection and reconstruction	65	32	(49.2)	13	(20.0)	6	(9.2)	2	(3.1)	3	(4.6)	3	(4.6)	12	(18.5)	1	(1.5)
(6) Robot-assisted esophagectomy and reconstruction	1415	743	(52.5)	303	(21.4)	81	(5.7)	34	(2.4)	80	(5.7)	134	(9.5)	166	(11.7)	13	(0.9)
(7) Others	24	9	(37.5)	3	(12.5)	1	(4.2)	0		1	(4.2)	3	(12.5)	0		0	
(8) Esophagectomy without reconstruction	102																
Subtotal	3426	1777	(51.9)	707	(20.6)	193	(5.6)	91	(2.7)	192	(5.6)	349	(10.2)	391	(11.4)	34	(1.0)
Total	5557	2814	(50.6)	1116	(20.1)	297	(5.3)	158	(2.8)	307	(5.5)	563	(10.1)	651	(11.7)	54	(1.0)

	Cases	Nonsurgical complications																					
		Pneumonia		Unplanned intubation		prolonged ventilation > 48 h		pulmonary embolism		atelectasis		Renal failure		CNS events		Cardiac events		Septic shock		Readmission within 30d		Reoperation within 30d	
Location																							
(1) Cervical esophagus	144	15	(10.4)	6	(4.2)	6	(4.2)	2	(1.4)	3	(2.1)	1	(0.7)	0		0		1	(0.7)	4	(2.8)	12	(8.3)
(2) Thoracic esophagus	4592	703	(15.3)	145	(3.2)	147	(3.2)	52	(1.1)	154	(3.4)	29	(0.6)	13	(0.3)	16	(0.3)	25	(0.5)	111	(2.4)	244	(5.3)
(3) Abdominal esophagus	565	72	(12.7)	11	(1.9)	16	(2.8)	5	(0.9)	24	(4.2)	1	(0.2)	3	(0.5)	4	(0.7)	2	(0.4)	12	(2.1)	28	(5.0)
Total	5301	790	(14.9)	162	(3.1)	169	(3.2)	59	(1.1)	181	(3.4)	31	(0.6)	16	(0.3)	20	(0.4)	28	(0.5)	127	(2.4)	284	(5.4)
Tumor depth																							
(A) Superficial cancer(T1)																							
(1) Transhiatal esophagectomy	3	0		0		0		0		0		0		0		0		0		0		0	
(2) Mediastinoscopic esophagectomy and reconstruction	88	16	(18.2)	4	(4.5)	4	(4.5)	1	(1.1)	2	(2.3)	0		1	(1.1)	0	(0.0)	0		2	(2.3)	4	(4.5)
(3) Transthoracic (rt.) esophagectomy and reconstruction	945	135	(14.3)	27	(2.9)	28	(3.0)	8	(0.8)	31	(3.3)	2	(0.2)	1	(0.1)	3	(0.3)	6	(0.6)	18	(1.9)	52	(5.5)
(4) Transthoracic (lt.) esophagectomy and reconstruction	12	0		0		0		0		0		0		1	(8.3)	0		0		0		1	(8.3)
(5) Cervical esophageal resection and reconstruction	8	0		1	(12.5)	1	(12.5)	0		0		0		0		0		0		0		0	
(6) Robot-assisted esophagectomy and reconstruction	906	118	(13.0)	24	(2.6)	21	(2.3)	13	(1.4)	27	(3.0)	5	(0.6)	2	(0.2)	2	(0.2)	3	(0.3)	29	(3.2)	42	(4.6)
(7) Others	14	1	(7.1)	1	(7.1)	1	(7.1)	0		0		0		0		0		0		0		0	
(8) Esophagectomy without reconstruction	155																						
Subtotal	2131	270	(12.7)	57	(2.7)	55	(2.6)	22	(1.0)	60	(2.8)	7	(0.3)	5	(0.2)	5	(0.2)	9	(0.4)	49	(2.3)	99	(4.6)
(B) Advanced cancer(T2-T4)																							
(1) Transhiatal esophagectomy	6	1	(16.7)	0		1	(16.7)	0		0		0		0		0		0		1	(16.7)	0	
(2) Mediastinoscopic esophagectomy and reconstruction	131	22	(16.8)	6	(4.6)	6	(4.6)	2	(1.5)	5	(3.8)	1	(0.8)	0		4	(3.1)	2	(1.5)	4	(3.1)	8	(6.1)
(3) Transthoracic (rt.) esophagectomy and reconstruction	1654	284	(17.2)	58	(3.5)	66	(4.0)	14	(0.8)	68	(4.1)	14	(0.8)	6	(0.4)	7	(0.4)	12	(0.7)	34	(2.1)	93	(5.6)
(4) Transthoracic (lt.) esophagectomy and reconstruction	29	5	(17.2)	1	(3.4)	2	(6.9)	1	(3.4)	1	(3.4)	2	(6.9)	0		0		0		3	(10.3)	2	(6.9)
(5) Cervical esophageal resection and reconstruction	65	5	(7.7)	3	(4.6)	4	(6.2)	1	(1.5)	2	(3.1)	0		0		0		0		2	(3.1)	5	(7.7)
(6) Robot-assisted esophagectomy and reconstruction	1415	200	(14.1)	36	(2.5)	35	(2.5)	19	(1.3)	44	(3.1)	7	(0.5)	5	(0.4)	4	(0.3)	5	(0.4)	34	(2.4)	77	(5.4)
(7) Others	24	2	(8.3)	0		0		0		1	(4.2)	0		0		0		0		0		0	
(8) Esophagectomy without reconstruction	102																						
Subtotal	3426	519	(15.1)	104	(3.0)	114	(3.3)	37	(1.1)	121	(3.5)	24	(0.7)	11	(0.3)	15	(0.4)	19	(0.6)	78	(2.3)	185	(5.4)
Total	5557	789	(14.2)	161	(2.9)	169	(3.0)	59	(1.1)	181	(3.3)	31	(0.6)	16	(0.3)	20	(0.4)	28	(0.5)	127	(2.3)	284	(5.1)

**Fig. 4:**
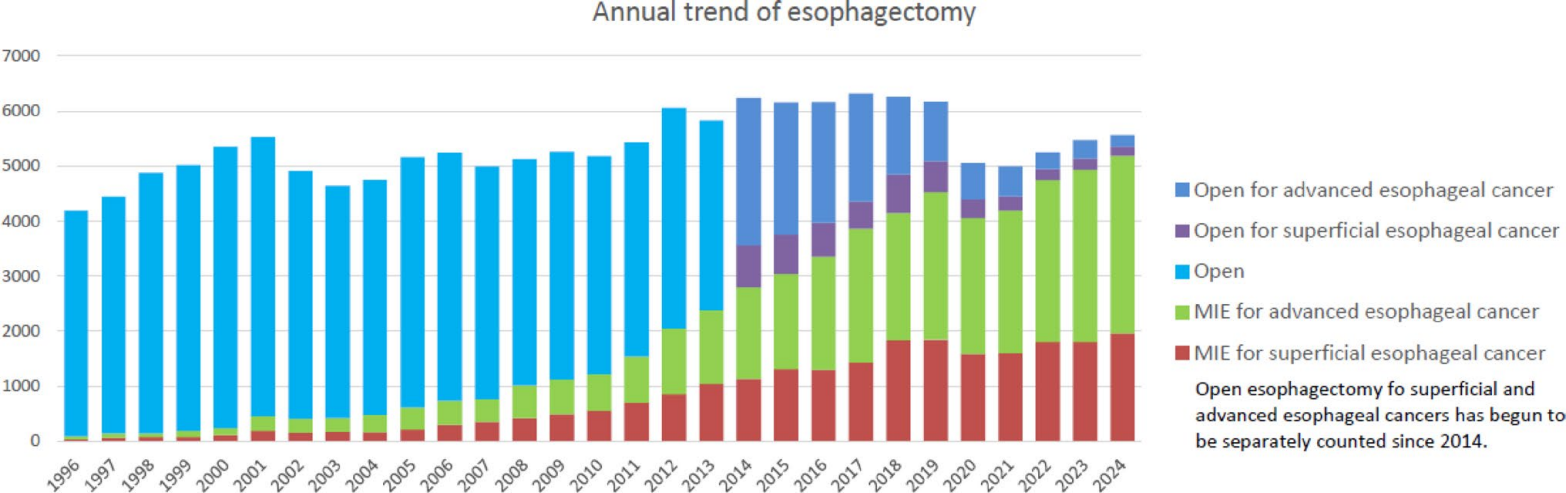
Annual trend of esophagectomy

Among esophagectomy procedures, transthoracic esophagectomy via right thoracotomy or right thoracoscopy was most commonly adopted for patients with superficial (945/2131, 44.3%) and advanced cancer (1654/3426, 48.3%) (Table 35). Transhiatal esophagectomy, which is commonly performed in Western countries, was adopted in only 3 (0.1%) and 6 (0.2%) patients with superficial and advanced cancer who underwent esophagectomy in Japan, respectively. Minimally invasive esophagectomy (MIE) including thoracoscopic and/or laparoscopic esophagectomy, robot-assisted esophagectomy and mediastinoscopic esophagectomy was utilized in 1967 (92.3%) and 3219 (94.0%) patients with superficial and advanced cancer, respectively. Incidence of MIE for superficial or advanced cancer have been increasing, whereas that of open surgery, especially for advanced cancer, has been decreasing annually (Fig. 4). Although mediastinoscopic esophagectomy was performed only for 88 (4.1%) and 131 (3.8%) patients with superficial and advanced esophageal cancer, respectively. Robot-assisted esophagectomy has been remarkably increased since 2018 when the insurance approval was obtained in Japan, and performed for 888 (41.7%) and 1398 (40.8%) patients with superficial and advanced esophageal cancer, respectively in 2024. The number of robot-assisted surgery in 2024 increased by 452 cases compared to 2023. Hospital mortality rates within 30 days after MIE were 0.4% and 0.8% for patients with superficial and advanced cancer, respectively (Table 35).

Detailed data collection regarding postoperative surgical and non-surgical complications after esophageal cancer surgery was initiated in 2018. Overall, 1116 (20.1%) of 5557 patients developed grade III or higher complications based on the Clavien–Dindo classification in 2024 (Table 35). The incidence of grade III or higher complications was relatively higher in cervical esophageal cancer compared to thoracic or abdominal esophageal cancer. Among surgical complications in patients with advanced esophageal cancer, anastomotic leakage and recurrent nerve palsy occurred in 11.2% and 11.1% of the patients who underwent right transthoracic esophagectomy, in 9.5% and 11.7% of those who underwent robot-assisted esophagectomy, and in 14.5% and 21.4% of those who underwent mediastinoscopic esophagectomy, respectively. Although the incidence of postoperative recurrent laryngeal nerve palsy in mediastinoscopic surgery has shown a tendency to improve year by year, it remained markedly higher than in other surgical approaches. Among non-surgical postoperative complications, pneumonia occurred in 14.2% of the patients, 2.9% of whom underwent unplanned intubation. Postoperative pulmonary embolism occurred in 1.1% of the patients. These complication rates, including the others, were similar to those in 2023.

We aim to continue our efforts in collecting comprehensive survey data through more active collaboration with the Japan Esophageal Society and other related institutions.

## Data Availability

Based on the data use policy of JATS, data access is approved through assessment by the JATS: Committee for Scientific Affairs. Those interested in using the data should contact the JATS: Committee for Scientific Affairs (survey@jpats.org) to submit a proposal. The use of the data is granted for the approved study proposals.
